# Immunogenicity and cross-protective efficacy induced by delayed attenuated *Salmonella* with regulated length of lipopolysaccharide in mice

**DOI:** 10.1080/19490976.2024.2424983

**Published:** 2024-11-11

**Authors:** Xiaoping Bian, Qing Liu, Yaolin Chen, Wenjin Zhang, Mengru Li, Xiaofen Zhang, Liu Yang, Yonghong Liao, Qingke Kong

**Affiliations:** a College of Veterinary Medicine, Southwest University, Beibei, Chongqing, China; b Yibin Academy of Southwest University, Yibin, Sichuan, China; c National Center of Technology Innovation for Pigs, Rongchang, Chongqing, China

**Keywords:** Regulated delayed attenuated *Salmonella*, outer membrane proteins, lipopolysaccharide, cross-protection, salmonella serotypes

## Abstract

Non-typhoidal *Salmonella enterica* (NTS) is a major global foodborne pathogen that poses a major public health concern worldwide, and no vaccines are available for protecting against infection of multiple *Salmonella* serotypes, therefore, the development of *Salmonella* vaccines to provide broad protection is valuable. In this work, we aimed to regulate lipopolysaccharide (LPS) synthesis of live *Salmonella in vivo* for exposing conserved protein antigens on the outer membrane while maintaining smooth LPS patterns *in vitro* to keep their original ability to invade host cells for inducing cross-protection against infection of multiple *Salmonella* serotypes. We generated a series of mutants defective in genes to affect the length of LPS. These mutants exhibit *in vivo* regulated-delayed attenuation and altered length of LPS, and all these mutants were derived from SW067 (Δ*pagL7* Δ*pagP81*::P_lpp_ *lpxE* Δ*lpxR9* Δ*fur9*) containing ∆*pagP81*::P_lpp_ *lpxE* mutation to reduce their endotoxic activity. Animal experiments demonstrated that all regulated delayed attenuated mutants exhibited reduced ability to colonize the organs of the mice, and SW114 (*waaI*), SW116 (*waaJ*), SW118 (*waaL*), and SW120 (*wbaP*) induced a significant production of IgG and IgA against OMPs isolated from *S*. Typhimurium, *S*. Enteritidis, and *S*. Choleraesuis. SW114 (*waaI*), SW116 (*waaJ*), and SW118 (*waaL*) were capable of conferring significant protection against infection of wild-type *S*. Enteritidis and *S*. Choleraesuis, with SW118 (*waaL*) triggering significant CD4^+^ T-cell responses as well as the B220^low^ IgG^+^ B_M_ cell. In conclusion, regulated delayed attenuated *Salmonella* vaccines with the whole core oligosaccharides of LPS showed a goo.d ability to expose conserved outer antigens and to trigger strong cross-immune responses against both homologous and heterologous *Salmonella* infections. These results give new insight into the development of the *Salmonella* vaccine against multiple serotypes of *Salmonella*.

## Introduction

*Salmonella enterica* is a significant zoonotic foodborne pathogen that causes considerable financial losses to the livestock industry and poses severe human health concerns worldwide.^1^ *Salmonella* is categorized into typhoidal *Salmonella* and non-typhoidal *Salmonella* (NTS) based on their ability to induce specific pathologies in humans.^2^ Typhoidal *Salmonella* is commonly known as Typhi (*Salmonella enterica* serovar Typhi) and Paratyphi (*Salmonella enterica* serovar Paratyphi) A, B, or C, whereas other serovars are referred to as NTS.^3^ According to the Global Burden of Disease 2021 study, a total of 9.32 million typhoid and paratyphoid cases were reported. Furthermore, there were 510 thousand cases of NTS invasive disease reported during the same period. In addition to enteric fever, which only occurs in human infection, NTS is a significant cause of global foodborne diseases through the transmission of *Salmonella* from animals to humans.^4^ Among the NTS serovars isolated from food, *Salmonella enterica* serovar Typhimurium (*S*. Typhimurium) was the most prevalent one in Africa,^5^ Australia,^6^ and China,^2^ and *S*. Enteritidis was the dominant serovar in the United States and European countries.^7,8^ Other serovars such as *S*. Anatum (*Salmonella enterica* serovar Anatum) and *S*. Weltevreden (*Salmonella enterica* serovar Weltevreden) were also frequently isolated in beef and seafood in the world^9^ and *S*. Derby, *S*. Thompson, and *S*. Aberdeen are the most common serotypes detected in chicken, pig, duck, aquatic products, and soft-shell turtle in China, respectively.^2^

An increasing trend of antimicrobial resistance (AMR) in *Salmonella*, especially multidrug resistance (MDR), has been on the rise and is a global concern.^10^ Vaccination has been demonstrated as a highly effective strategy to combat infections of antibiotic-resistant *Salmonella*. *Salmonella* vaccines that are currently authorized for use in humans selectively target *S*. Typhi, and three different types of approved typhoidal *Salmonella* vaccines are available, including the live attenuated oral vaccine Ty21a, the unconjugated Vi polysaccharide vaccine, and the Typhoid conjugated vaccine (TCV).^11^ Since these vaccines primarily target *Salmonella* surface polysaccharide antigens, there is limited cross-protection against other serotypes of *Salmonella*, although Ty21a, derived from *S*. Typhi, confers moderate cross-protection against *S*. Paratyphi B.^12^ At present, there are no licensed vaccines against *Salmonella* Paratyphi A, B, or C, which are responsible for causing paratyphoid fever, and no NTS vaccines available for humans, while the use of NST vaccines for animals is limited.^13^ For human NTS vaccine development, several vaccine developers prefer to develop combination vaccines addressing important *Salmonella* serotypes, such as the trivalent combination vaccine, which is formulated with *Salmonella* Typhi plus *S*. Enteritidis in serogroup D carrying O:9 antigen and *S*. Typhimurium in serogroup B carrying O:4 antigen and is meant to cover the predominant circulating serovars in sub-Saharan Africa and Asia.^14^ *Salmonella* vaccines for animals are mainly used to prevent fowl typhoid caused by *S*. Gallinarum of serogroup D carrying the O:9 antigen, infections in laying hens caused by *S*. Typhimurium and Enteritidis, and piglet paratyphoid fever caused by *S*. Choleraesuis of serogroup C carrying the O:7 antigen.^15,16^ In general, no reports have systematically addressed the efficacy of monovalent NTS vaccine formulations across serogroups, and more importantly, it is necessary to develop a monovalent, broad-spectrum NTS vaccine that provides broad cross-protection against multiple NTS.

Although the millions of lipopolysaccharide (LPS) molecules present on the surface of *Salmonella* create a formidable barrier to restrict antibody access to the bacterial surface,^17^ previous studies have demonstrated that isolated outer membrane proteins (OMPs) from *Salmonella* conferred protection against both homologous and heterologous serotype *Salmonella* challenges in mice,^17–20^ indicating that certain OMPs are highly conserved in *Salmonella* to confer cross-protection. These studies imply that the OMPs of *Salmonella* are promising targets for the development of vaccines against infection with multiple serotypes. The process of purifying OMPs from *Salmonella* is both labor-intensive and costly, and some conserved OMPs are low or not expressed under laboratory conditions, rendering them unsuitable for large-scale production and application. Hence, live attenuated *Salmonella* vaccines may be ideal candidates for developing a vaccine with broad protection due to their cost-effectiveness, ease of management, and the capacity to generate long-lasting immunity with just one dosage. Our earlier research has shown that while attenuated *Salmonella* with permanent truncated LPS induces somewhat cross-immune responses against other *Salmonella* serotypes and enteric bacteria, mutants do not effectively colonize the host intestine to induce robust immune responses.^19,20^ Therefore, attenuated *Salmonella* with the permanent truncated LPS is not a suitable candidate for vaccine development.

Regulated delayed attenuated *Salmonella* (RDAS) that was developed in Curtiss’s lab possesses the critical feature of undergoing regulated delayed attenuation *in vivo*. RDAS vaccine candidates are allowed to synthesize virulence factors required for colonization and invasion by adding specific substances to the culture medium *in vitro*, and after colonization in the organs, the mutants gradually undergo attenuation due to the absence of specific inducers after oral immunization *in vivo*.^21,22^ Therefore, we wanted to investigate whether the regulated length of LPS in attenuated *Salmonella* would expose more conserved antigens, thus inducing more effective cross-immunity. In this study, we constructed a series of RDAS that regulate LPS synthesis, which is capable of synthesizing full LPS lengths in the presence of arabinose *in vitro* while synthesizing different truncated lengths of LPS in the absence of arabinose *in vivo*.^23^ Our objective was to develop ideal candidates for live attenuated *Salmonella* vaccines to trigger cross-immune responses effectively and confer cross-protection against infection of multiple serotypes of *Salmonella*.

## Material and methods

### Growth conditions, medium, plasmids, and strains of bacteria

Table 1 provides a comprehensive list of all the strains of bacteria and plasmids used in this study. *Salmonella* and *E. coli* were routinely cultivated either in Luria-Bertani (LB) broth (L1010, Solarbio Biotech, China) or on LB agar (L1015, Solarbio Biotech, China)^30^ with or without 0.1% arabinose at 37°C. In cases where necessary, the culture media were supplemented with 25 μg/ml of chloramphenicol (A600118, Sangon Biotech, China). To facilitate the growth of Asd- strains, 2,6-diaminopimelic acid (DAP) was included at a final concentration of 50 µg/ml (D1377, Sigma Aldrich, USA).^31^ For the cultivation of strains involving allelic exchange experiments, *sacB* gene-based counterselection was conducted on LB agar containing 5% sucrose (A610498, Sangon Biotech, China). All *Salmonella* mutants originated from the *S*. Typhimurium strain UK-1,^24^ and their genotypes were verified through PCR employing the corresponding primer pair outlined in Table 2.

**Table 1.** Bacterial strains and plasmids used in this study.

Strains or plasmids	Descriptions				Source
***Salmonella*****strains**					
χ3761	Wild type *S*. Typhimurium, UK-1				24
χ3700	*S*. Enteritidis, a clinical isolate from chicken, S246				20
χ3545	*S*. Choleraesuis, a clinical isolate from pig, S340				20
χ9705	Δ*pagL7* Δ*pagP81*::P_lpp_ *lpxE* Δ*lpxR9*				25
SW067	χ9705 Δ*fur9*				Lab stock
SW079	Δ*waaC40*				This study
SW080	Δ*waaF41*				This study
SW081	Δ*waaG42*, the same strain as χ11308				26
SW082	Δ*waaI43*, the same strain as χ11309				26
SW083	Δ*waaJ44*, the same strain as χ11310				26
SW084	Δ*waaL46*, the same strain as χ11312				26
SW085	Δ*wbaP45*, the same strain as χ11311				26
SW086	Δ*waaC40* Δ*pagL7*				This study
SW087	Δ*waaF41* Δ*pagL7*				This study
SW088	Δ*waaG42* Δ*pagL7*				This study
SW089	Δ*waaI43* Δ*pagL7*				This study
SW090	Δ*waaJ44* Δ*pagL7*				This study
SW091	Δ*waaL46* Δ*pagL7*				This study
SW092	Δ*wbaP45* Δ*pagL7*				This study
SW093	Δ*waaC40* Δ*pagL71*::TT *araC* P_BAD_ *waaC*				This study
SW094	Δ*waaC40* Δ*pagL72*::TT *araC* P_BAD_ *waaC*				This study
SW095	Δ*waaF41* Δ*pagL73*::TT *araC* P_BAD_ *waaF*				This study
SW096	Δ*waaF41* Δ*pagL74*::TT *araC* P_BAD_ *waaF*				This study
SW097	Δ*waaG42* Δ*pagL75*::TT *araC* P_BAD_ *waaG*				This study
SW098	Δ*waaG42* Δ*pagL76*::TT *araC* P_BAD_ *waaG*				This study
SW099	Δ*waaI43* Δ*pagL77*::TT *araC* P_BAD_ *waaI*				This study
SW100	Δ*waaI43* Δ*pagL78*::TT *araC* P_BAD_ *waaI*				This study
SW101	Δ*waaJ44* Δ*pagL79*::TT *araC* P_BAD_ *waaJ*				This study
SW102	Δ*waaJ44* Δ*pagL80*::TT *araC* P_BAD_ *waaJ*				This study
SW103	Δ*waaL46* Δ*pagL81*::TT *araC* P_BAD_ *waaL*				This study
SW104	Δ*waaL46* Δ*pagL82*::TT *araC* P_BAD_ *waaL*				This study
SW105	Δ*wbaP45* Δ*pagL83*::TT *araC* P_BAD_ Δ*wbaP*				This study
SW106	Δ*wbaP45* Δ*pagL84*::TT *araC* P_BAD_ Δ*wbaP*				This study
SW107	Δ*pagL7* Δ*pagP81*::P_lpp_ *lpxE* Δ*lpxR9* Δ*fur9* ∆*waaC40*				This study
SW108	Δ*pagL71*::TT *araC* P_BAD_ *waaC* Δ*pagP81*::P_lpp_ *lpxE* Δ*lpxR9* Δ*fur9* ∆*waaC40*				This study
SW109	Δ*pagL7* Δ*pagP81*::P_lpp_ *lpxE* Δ*lpxR9* Δ*fur9* ∆*waaF41*				This study
SW110	Δ*pagL74*::TT *araC* P_BAD_ *waaF* Δ*pagP81*::P_lpp_ *lpxE* Δ*lpxR9* Δ*fur9* ∆*waaF41*				This study
SW111	Δ*pagL7* Δ*pagP81*::P_lpp_ *lpxE* Δ*lpxR9* Δ*fur9* ∆*waaG42*				This study
SW112	Δ*pagL76*::TT *araC* P_BAD_ *waaG* Δ*pagP81*::P_lpp_ *lpxE* Δ*lpxR9* Δ*fur9* ∆*waaG42*				This study
SW113	Δ*pagL7* Δ*lpxR9* Δ*pagP81*::P_lpp_ *lpxE* Δ*fur9 ∆waaI43*				This study
SW114	Δ*pagL77*::TT *araC* P_BAD_ *waaI* Δ*pagP81*::P_lpp_ *lpxE* Δ*lpxR9* Δ*fur9* ∆*waaI43*				This study
SW115	Δ*pagL7* Δ*pagP81*::P_lpp_ *lpxE* Δ*lpxR9* Δ*fur9* ∆*waaJ44*				This study
SW116	Δ*pagL79*::TT *araC* P_BAD_ *waaJ* Δ*pagP81*::P_lpp_ *lpxE* Δ*lpxR9* Δ*fur9* ∆*waaJ44*				This study
SW117	Δ*pagL7* Δ*pagP81*::P_lpp_ *lpxE* Δ*lpxR9* Δ*fur9* ∆*waaL46*				This study
SW118	Δ*pagL81*::TT *araC* P_BAD_ *waaL* Δ*pagP81*::P_lpp_ *lpxE* Δ*lpxR9* Δ*fur9* ∆*waaL46*				This study
SW119	Δ*pagL7* Δ*pagP81*::P_lpp_ *lpxE* Δ*lpxR9* Δ*fur9* ∆*wbaP45*				This study
SW120	Δ*pagL83*::TT *araC* P_BAD_ Δ*wbaP* Δ*pagP81*::P_lpp_ *lpxE* Δ*lpxR9* Δ*fur9* ∆*wbaP45*				This study
***E. coli*****strains**					
χ7232	*endA1 hsdR17* (*r*_*K*_*-, m*_*k*_*+*) *glnV44 thi-1 recA1 gyrA relA1* ∆(*lacZYA-argF*)*U169 λ*pir *deoR* (ϕ*80dlac* ∆(*lacZ*)*M15*)			27	
χ7213	*thi-1 thr-1 leuB6 glnV44 fhuA21 lacY1 recA1* RP4-2-Tc:Mu[*λ* pir] ∆*asdA4* ∆(*zhf-2*::Tn*10*)			27	
**Plasmids**					
pYA4278	*sacB mobRP4* R6K *ori* Cm+, derived from pRE112			28	
pYA3700	Cloning vector containing TT *araC* P_BAD_ cassette			29	
pYA4284	∆*pagL7*			25	
pSW012	for ∆*waaC40* mutation, suicide plasmid			This study	
pSW013	for Δ*waaF41* mutation, suicide plasmid			This study	
pSW014	for Δ*waaG42* mutation, the same suicide plasmid as pYA4896			26	
pSW015	for Δ*waaI43* mutation, the same suicide plasmid as pYA4897			26	
pSW016	for Δ*waaJ44* mutation, the same suicide plasmid as pYA4898			26	
pSW017	for Δ*waaL46* mutation, the same suicide plasmid as pYA4900			26	
pSW018	for Δ*wbaP45* mutation, the same suicide plasmid as pYA4899			26	
pSW019	Insert *waaC1* gene to pYA3700			This study	
pSW020	Insert *waaC2* gene to pYA3700			This study	
pSW021	Insert *waaF1* gene to pYA3700			This study	
pSW022	Insert *waaF2* gene to pYA3700			This study	
pSW023	Insert *waaG1* gene to pYA3700			This study	
pSW024	Insert *waaG2* gene to pYA3700			This study	
pSW025	Insert *waaI1* gene to pYA3700			This study	
pSW026	Insert *waaI2* gene to pYA3700			This study	
pSW027	Insert *waaJ1* gene to pYA3700			This study	
pSW028	Insert *waaJ2* gene to pYA3700			This study	
pSW029	Insert *waaL1* gene to pYA3700			This study	
pSW030	Insert *waaL2* gene to pYA3700			This study	
pSW031	Insert *wbaP1* gene to pYA3700			This study	
pSW032	Insert *wbaP2* gene to pYA3700			This study	
pSW033	for Δ*pagL71*::TT *araC* P_BAD_ *waaC* mutation			This study	
pSW034	for Δ*pagL72*::TT *araC* P_BAD_ *waaC* mutation			This study	
pSW035	for Δ*pagL73*::TT *araC* P_BAD_ *waaF* mutation			This study	
pSW036	for Δ*pagL74*::TT *araC* P_BAD_ *waaF* mutation			This study	
pSW037	for Δ*pagL75*::TT *araC* P_BAD_ *waaG* mutation			This study	
pSW038	for Δ*pagL76*::TT *araC* P_BAD_ *waaG* mutation			This study	
pSW039	for Δ*pagL77*::TT *araC* P_BAD_ *waaI* mutation			This study	
pSW040	for Δ*pagL78*::TT *araC* P_BAD_ *waaI* mutation			This study	
pSW041	for Δ*pagL79*::TT *araC* P_BAD_ *waaJ* mutation			This study	
pSW042	for Δ*pagL80*::TT *araC* P_BAD_ *waaJ* mutation			This study	
pSW043	for Δ*pagL81*::TT *araC* P_BAD_ *waaL* mutation			This study	
pSW044	for Δ*pagL82*::TT *araC* P_BAD_ *waaL* mutation			This study	
pSW045	for Δ*pagL83*::TT *araC* P_BAD_ *wbaP* mutation			This study	
pSW046	for Δ*pagL84*::TT *araC* P_BAD_ *wbaP* mutation			This study

**Table 2.** Prime used in this study.

Primer name	Sequence (5’-3’) (the sequences highlighted by red color contain SD and start codon)
WaaC-Del 1F	cattctgaaatgagccggcgctgaatagcgagcag
WaaC-Del 1 R	cagagtctctttaaacgccctcttccgaca
WaaC-Del 2F	gggcgtttaaagagactctgtctcatccca
WaaC-Del 2 R	agcatttatcagggtaagccctccagtaccgtatt
Vec-F	accctgataaatgcttcaataa
Vec-R	gctcatttcagaatggaaggtc
WaaF-Del 1F	cattctgaaatgagcgaaagcccgaaactgtttga
WaaF-Del 1 R	aaacccgcatacttacgcgtcgcggttcag
WaaF-Del 2F	acgcgtaagtatgcgggttttgatcgttaa
WaaF-Del 2 R	agcatttatcagggtctccagtccataccgtgctttat
WaaC-1F	tttctccataaaggctctatatgcgggttttgatcgttaa
WaaC-2F	tttctccataaaggctctatgtgcgggttttgatcgttaa
WaaC-R	aaaaaacgggttaatgaatctttccaaatac
WaaC-VF	gattcattaacccgtttttttgggctagcc
WaaC-VR	atagagcctttatggagaaacagtagagagt
WaaF-1F	gtttctccataaaggctctatatgaaaattttggtcattggc
WaaF-2F	gtttctccataaaggctctatgtgaaaattttggtcattggc
WaaF-R	caaaaaaacgggttaaacgccctcttccgacaa
WaaF-VF	gggcgtttaacccgtttttttgggctagcc
WaaF-VR	atagagcctttatggagaaacagtagagagt
WaaG-1F	gtttctccataaaggctctatatgagagttgccttttgcttat
WaaG-2F	gtttctccataaaggctctatgtgagagttgccttttgcttat
WaaG-R	caaaaaaacgggtcaaccatctaaatcacctg
WaaG-VF	agatggttgacccgtttttttgggctagcc
WaaG-VR	atagagcctttatggagaaacagtagagagt
WaaI-1F	gtttctccataaaggctctatatgagcagaaaatattttgaag
WaaI-2F	gtttctccataaaggctctatgtgagcagaaaatattttgaag
WaaI-R	aaaaaacgggttattcaagaagtttacgttt
WaaI-VF	cttgaataacccgtttttttgggctagcc
WaaI-VR	atagagcctttatggagaaacagtagagagt
WaaJ-1F	gtttctccataaaggctctatatggattcatttcctgagata
WaaJ-2F	gtttctccataaaggctctatgtggattcatttcctgagata
WaaJ-R	aaaaaacgggttatttgtggaaaagtttac
WaaJ-VF	ccacaaataacccgtttttttgggctagcc
WaaJ-VR	atagagcctttatggagaaacagtagagagt
WaaL-1F	gtttctccataaaggctctatatgctaaccacatcattaac
WaaL-2F	gtttctccataaaggctctatgtgctaaccacatcattaac
WaaL-R	aaaaaacgggttatctatttcttagcgccag
WaaL-VF	gaaatagataacccgtttttttgggctagcc
WaaL-VR	atagagcctttatggagaaacagtagagagt
WbaP −1F	gtttctccataaaggctctatatggttgagctgaaagcgccg
WbaP −2F	gtttctccataaaggctctatgtggttgagctgaaagcgccg
WbaP -R	caaaaaaacgggttacagattttttcttattg
WbaP -VF	aaatctgtaacccgtttttttgggctagcc
WbaP -VR	atagagcctttatggagaaacagtagagagt
P1	ttgaaatggtggtggatttattattctatcctagaat
P2-(WaaC)	aattgttattcaactttaatgaatctttccaaatac
p-VF	agttgaataacaattagcgag
p-VR	tccaccaccatttcaatgtcaa
P3-(WaaF)	ctaattgttattcaactttaaacgccctcttccgacaa
P4-(WaaG)	ctaattgttattcaacttcaaccatctaaatcacctgt
P5-(WaaI)	ctaattgttattcaactttattcaagaagtttacgttt
P6-(WaaJ)	taattgttattcaactttatttgtggaaaagtttacg
P7-(WaaL)	taattgttattcaactttatctatttcttagcgccag
P8-(WbaP)	aattgttattcaactttacagattttttcttattgtc

### Construction of plasmids and mutant strains

The allelic exchange method was utilized to introduce gene mutations in *Salmonella*, employing pYA4278 as described in the previous study.^28^ The transformation of *E. coli* was carried out using a technique known as electroporation. To select the transformants, appropriate antibiotic supplements were added to LB agar plate cultures. Primers utilized in this study were listed in Table 2. For arabinose-regulated LPS synthesis in this study, we applied the same strategy as the Δ*pagL*::TT *araC* P_BAD_ *wbaP* deletion-insertion mutation to construct other mutations.^32^ Briefly, for the construction of the *waaC* mutation, in which the complete *waaC* open-reading frame was deleted, the χ3761 genomes were employed as the template for cloning, a DNA fragment of 350-bp containing the region upstream of the *waaC* gene (from ATG start codon, but not including ATG) was amplified using primers WaaC-Del 1F and WaaC-Del 1 R, and another 350-bp DNA fragment containing the region downstream of the *waaC* gene (from TAA stop codon, but not including TAA) was amplified using primers WaaC-Del 2F and WaaC-Del 2 R. Universal primers Vec-F and Vec-R were applied to amplify vector fragments. The 5′ flanking regions of these DNA fragments contain homologous regions for recombination. The PCR products were purified by agarose gel (A620014, Sangon Biotech, China) and ligated to pYA4278 using Gibson Assembly Master Mix (E55510, New England Biolabs, USA) to generate the suicide plasmid pSW012 for deleting the *waaC* gene. The *waaC* mutation was introduced into χ3761 and SW067 by allelic exchange via conjugation with the χ7213, harboring the plasmid of pSW012, to generate SW079 (∆*waaC40*) and SW107 (Δ*pagL7* Δ*pagP81*::P_lpp_ *lpxE* Δ*lpxR9* Δ*fur9* ∆*waaC40*), respectively. The genotypes of Δ*waaC* were verified by PCR and LPS profiles. The same strategy also applied to the deletion of the *waaF*, *waaG*, *waaI*, *waaJ*, *waaL*, or *wbaP* genes in the χ3761 and SW067 genomes (Table 1).

For the construction of the Δ*pagL*::TT *araC* P_BAD_ *waaC* deletion-insertion mutation, the vector of pYA3700^29^ was linearized using the primers WaaC-VF and WaaC-VR. The fragment *waaC* gene was then amplified by WaaC-1F/WaaC-R and WaaC-2F/WaaC-R from the χ3761 genomes and inserted into pYA3700 via Gibson Assembly Master Mix to generate intermediate plasmid pSW019 and pSW020, respectively. Subsequently, the fragment TT *araC* P_BAD_ *waaC* was amplified using primers P1 and P2-(WaaC), and the gel-purified product was ligated to the linearized vector pYA4284 by Gibson Assembly Master Mix to form the suicide plasmids pSW033 and pSW034, respectively. The *pagL* mutation was introduced into SW079 (Δ*waaC40*) by allelic exchange via conjugation with the χ7213 harboring the plasmid of pYA4284 to yield the strain SW086 (Δ*waaC40* Δ*pagL7*). The Δ*pagL*::TT *araC* P_BAD_ *waaC* mutation was introduced into SW086 by allelic exchange by conjugation with the χ7213 harboring the plasmids of pSW033 or pSW034 to yield SW093 (∆*waaC40* Δ*pagL71*::TT *araC* P_BAD_ *waaC*) or SW094 (∆*waaC40* Δ*pagL72*::TT *araC* P_BAD_ *waaC*), respectively. PCR and LPS profiles were applied to confirm the genotypes of the Δ*pagL*::TT *araC* P_BAD_ *waaC* mutations. After the mutation with tightly regulated LPS was successfully screened and identified in the wild-type background, the correct mutation was introduced into the mutant strain SW107 (Δ*pagL7* Δ*pagP81*::P_lpp_ *lpxE* Δ*lpxR9* Δ*fur9* ∆*waaC40*) to generate the mutant strain SW108 (Δ*pagL71*::TT *araC* P_BAD_ *waaC* Δ*pagP81*::P_lpp_ *lpxE* Δ*lpxR9* Δ*fur9* ∆*waaC40*). The other mutant strains were generated using the same procedure.

### Phenotypic determination of bacteria

Bacterial strain phenotypes were identified *in vitro*, and each experiment was conducted at least twice. The OMPs and LPS were purified from *Salmonella* following described methods.^33–35^ The OMPs’ total protein concentrations were determined utilizing a bicinchoninic acid protein assay (ZJ101, Yazyme, China), following the guidelines provided by the manufacturer. The OMPs were separated using a 12.5% sodium dodecyl sulfate-polyacrylamide gel (SDS-PAGE) and subsequently stained with Coomassie brilliant blue (2 μg of OMPs in each lane). The LPS samples of *S*. Typhimurium underwent silver staining to establish their LPS profile after separation in 12.5% SDS-PAGE (2 μg of LPS in each lane), following the method described by Hitchcock and Brown.^36^ Lipid A was extracted from *Salmonella* after mild acid hydrolysis at pH 4.5 to break the Kdo-lipid A linkage and subjected to Matrix-Assisted Laser Desorption Ionization-Time of Flight Mass Spectrometry (MALDI-TOF MS).^37,38^

### Animal experimental studies

#### Mice

The 7-week-old female BALB/c mice used for the experiment were purchased from Hunan SJA Laboratory Animal Co., Ltd. All animal experiments adhered to the rules specified in the Guide for the Care and Use of Laboratory Animals. Conscientious efforts made an effort to avoid animal suffering during the experiments. All animals arrive and are given a seven-day acclimatization period before the start of the experiment.

#### Immunization of mice

Oral administration was used to immunize mice in this study. In summary, a 3 ml LB broth with 0.1% arabinose was chosen to cultivate delayed attenuated *Salmonella* at 37°C. On the next day, the cultures were adjusted by a ratio of 1:100 using a 100-ml medium of the same composition and then placed in an incubator under the same culture conditions until the optical density value of the bacterial solution at a wavelength of 600 nm (OD_600_) reached 0.85, which is approximately equivalent to 1 × 10^9^ CFU/ml. The bacterial strains were harvested by centrifuging a 100 ml suspension at 5000 rpm at the ambient temperature. The obtained bacterial pellet was then suspended in 2 ml of buffered saline with gelatin (48722, Millipore, USA) (BSG), which is approximately equivalent to 1 × 10^9^ CFU/20 µl. Prior to immunization, all animals underwent a 6-hour period of food and water fasting. Subsequently, all mice were administered a 20 µl oral dose of BSG solution or BSG containing 1 × 10^9^ CFU of the matching delayed attenuated *Salmonella*. For immunogenicity assessment and protective efficacy with delayed attenuated *Salmonella*, mice were booster-immunized 4 weeks after initial immunization. The researchers closely monitored the vaccinated mice for abnormalities, changes in overall health, as well as reduced food intake. In addition, any signs of disheveled fur, diarrhea, illness, and death are documented.

#### Bacterial colonization in organs

To determine the bacterial load in the tissues, three mice from every group were sacrificed at specific time points following one inoculation. Samples from the spleen and liver were obtained aseptically in an ultra-clean workbench and weighed individually. Peyer’s patches were obtained from all regions of the small intestine surface in an ultra-clean workbench, and the bacterial colonization in Peyer’s patches reflected the average bacterial load in combined Peyer’s patches from each animal. Each sample was added to 1 ml of phosphate buffer saline (PBS) to get a homogenized sample. Tissue homogenate was properly diluted in PBS and dropped on MacConkey agar and LB agar plates, and the number of bacteria on each plate was counted the following day. MacConkey agar plates were created specifically to eliminate the possible presence of *E. coli* in Peyer’s patches from affecting results. It is worth noting that 100 µl of undiluted tissue homogenates also need to be dropped onto MacConkey agar and LB agar. If no colonies were observed on the plate after culturing the 0.1 ml undiluted homogenizing of the organ, the selenite cysteine broth (100212, Sigma, USA) was used to inoculate the remaining tissue homogenates from each tissue sample the following day. Samples that showed positive results after being enriched in selenite cysteine broth at 37°C overnight were noted to have a CFU count of less than 10 per gram. The minimum detection limit in the bacterial burden test is < 10 CFU/g.

#### Bacterial loads in feces

To investigate the bacterial load in feces, mice were put individually in clean cages without any bedding on days 3, 6, 14, and 21 following a single inoculation, and a total of 4–6 fecal pellets were collected in a 2-ml tube. Fecal pellets were weighed and homogenized in PBS (0.1 g/ml). The quantification of bacteria per 100 mg of feces was performed by dropping serially diluted or undiluted fecal homogenate on an XLD agar (HB4105–6, Haibo, China) plate. The minimum detection limit in the bacterial burden test is < 5 CFU/100 mg.

#### Histology and pathological scores

Three mice per group were sacrificed on the seventh day after immunization and the liver and spleen were subsequently gathered to measure the extent of tissue damage induced by the delayed attenuated *Salmonella* according to the standard H&E staining method on paraffin-embedded tissues.^39^ Two researchers, unaware of group-specific data, assessed individual liver or spleen tissue samples from each group histopathologically. This evaluation was carried out following the five-level grading system scoring criteria in the International Harmonization of Nomenclature and Diagnostic Criteria for Lesions in Rats and Mice (INHAND), including hepatocyte vacuolization (0–4), necrosis with inflammatory cell infiltration in the liver tissue (0–4), extramedullary hematopoiesis in the spleen (0–4), necrosis with inflammatory cell infiltration in the spleen tissue (0–4), and splenic congestion (0–4). A score of 0 indicates normal limits, a score of 1 indicates slight tissue abnormality, a score of 2 indicates mild tissue abnormality, a score of 3 indicates moderate tissue abnormality and a score of 4 indicates severe tissue damage. The maximum cumulative histopathology score was 8 for each liver sample and 12 for each spleen sample. Mice orally administered wild-type *S*. Typhimurium χ3761 or BSG served as positive and negative controls for tissue damage, respectively.

#### Immunogenicity of vaccine strains in mice

For the assessment of immunogenicity, 64 mice were randomly divided into 8 groups (8 mice per group), including 7 experimental groups and one control group. Four weeks after the primary immunization, animals were administered an additional dose of the same delayed attenuated *Salmonella* or BSG that was identical to the prior dosage, and serum and vaginal secretion samples were collected at week 4 after the second immunization and antibody levels in the samples were measured by ELISA.

#### Survival assay

Sixty-four mice were randomized into 8 groups for assessment of the protective efficacy of immunization with the regulated attenuated *Salmonella* mutants at 9 weeks post-initial immunization (week 5 after second immunization) by oral challenge with 10^9^ CFU of wild-type *S*. Typhimurium (χ3761) in 20 μl BSG (~10000 × LD_50_).^19^ Furthermore, we also evaluated cross-protection conferred by regulated delayed attenuated *Salmonella* in another independent experiment. Sixteen groups of BALB/c mice (with 8 mice per group) were used to immunize, including fourteen experimental groups and two control groups. Mice were orally challenged with 10^7^ CFU/20 μl of *S*. Enteritidis (χ3700) or *S*. Choleraesuis (χ3545) at 9 weeks after the first immunization (week 5 after the second immunization), respectively. The χ3700 and χ3545 were clinical isolates from the chicken and pig, respectively, with an LD_50_ of 10^5^ CFU/20 μl.^19^ Challenged mice were monitored daily for evidence of unkempt fur, restlessness, diarrhea, morbidity, and mortality, which were recorded for 25 days after the challenge.

### ELISA sample collection

A mandibular vein puncture was performed to collect blood samples from mice. The serum was isolated from the blood cells using centrifugation at a speed of 3500 rpm for a total of 15 minutes, then aspirated with as much of the supernatant as possible, transferred to a clean 0.5-ml tube, and centrifuged again at 3500 rpm for 5 minutes. The supernatant was once again gathered into a 0.5-ml tube and then preserved at a temperature of −20°C if it was needed. To examine the IgA in mice elicited by regulated delayed attenuated *Salmonella*, the vaginal canal of each mouse was rinsed with 60 µl of TBS at specific time points, and the vaginal wash was collected in 0.5 ml tubes. The vaginal wash was kept at low temperatures throughout the entire process and harvested as it is used, avoiding repeated freezing and thawing.

### ELISA

The enzyme-linked immunosorbent assay (ELISA) was utilized to quantify the concentrations of antibodies specific to *Salmonella* LPS or OMPs in serum and vaginal wash samples.^40,41^ The *Salmonella* LPS or OMPs were coated onto an ELISA plate (3590, Corning Costar, USA) at a concentration of 200 ng/100 µl overnight under a temperature of around 4°C. To establish a standard curve for each antibody isotype, the mouse Ig standard (1010–01, Southern Biotech, USA) was coated onto the last two rows of each plate at a concentration of 200 ng/100 µl overnight under a temperature around 4°C. The plates underwent three wash cycles using TBS-0.1% Tween 20 before being blocked with 3% BSA at 37°C over 2 hours. Following the blocking process, all plates were washed three times with TBS-0.1% Tween 20. The serum and vaginal wash samples were diluted in TBS-1% BSA and added as primary antibodies to the corresponding enzyme plates at 37°C for 1 hour. The unconjugated anti-mouse IgG, IgA, IgG1, and IgG2a were used as primary antibodies for the standard curve in TBS-1% BSA in a stepwise manner by a factor of 2 manners. The IgG was diluted to a concentration of 0.488 ng/ml from a concentration of 500 ng/ml. Similarly, the concentrations of IgG1 and IgG2a were diluted from 1 µg/ml to 8 ng/ml. The IgA concentration was also diluted from 500 ng/ml to 0.488 ng/ml. Subsequently washing with TBS-0.1% Tween 20, a 1:5000 dilution of biotinylated goat anti-mouse IgA (1040–08, Southern Biotech, USA), IgG1 (1070–08, Southern Biotech, USA), IgG2a (1080–08, Southern Biotech, USA), or IgG (1030–08, Southern Biotech, USA) was given to corresponding well. The plates were thereafter placed at 37°C for an hour and washed three times at the end of the incubation. All plates were exposed to a streptavidin-alkaline phosphatase conjugate (7100–04, Southern Biotech, USA) diluted at a ratio of 1:3000 and incubated for an hour at 37°C. After that, all plates were washed three times, and a 100 µl solution of p-nitrophenyl phosphate (N1891, Sigma, USA) at a concentration of 1 mg/ml was added to each plate for a color development time of approximately 10 minutes at 37°C. Finally, 50 μL 3 M sodium hydroxide (A36865, Innochem, China) was added to terminate the color reaction and the absorbance of color development was measured for 405 nm with a Microplate Detector (1530, Thermo Fisher Scientific, USA). For the detection of serum and vaginal wash samples, the antigens for the coating plate were LPS or OMPs derived from *Salmonella* or *E. coli* and the dilution of the sample is 1:10 or 1:100. Through linear regression analysis in Excel with a coefficient of determination (R^2^) equal to or greater than 0.98, the OD_405_ was graphed versus the known levels of the diluted unconjugated antibody solutions to create standard curves. These standard curves were then applied to calculate the final concentrations of antibodies present in the samples.

### Complement deposition assays

Serum samples from the same group of mice previously collected at the 4th week after the second immunization were mixed and heat-inactivated at 56°C for 30 minutes. A 1 × 10^7^ CFU/100 µl wild-type *S*. Typhimurium (χ3761), *S*. Enteritidis (χ3700), or *S*. Choleraesuis (χ3545) strain was resuspended in 80 µl of PBS with 1% BSA and mixed with 20 µl of heat-inactivated pooled serum from the relevant group. The mixtures were then incubated at 37°C for 40 minutes. Subsequently, the treated *Salmonella* cells were washed twice with PBS and exposed to 100 µl of PBS-1% BSA solution containing 50 µl of rabbit complement (CL3441-R, Cedarlane, Canada) and incubated at 37°C for 30 minutes. The treated *Salmonella* strain in the previous step was washed twice with PBS, and then the *Salmonella* strain was stained with FITC-conjugated goat anti-rabbit complement polyclonal antibody (ab182878, Abcam, UK) at a final concentration of 1:100 in PBS with 1% BSA on ice for 25 minutes. Finally, the strain was resuspended in 500 µl of 2% paraformaldehyde after two washes with PBS, and the samples were examined using flow cytometry. Positive control sera are polyclonal antibodies against *Salmonella* O4, O7, or O9 O-antigen, and sera for negative controls were obtained from mice injected with BSG.

### Flow cytometry

Mice were euthanized 38 days after the initial immunization (10 days after the second vaccination), with 3 mice per group. The spleens were then collected aseptically on a clean bench. Spleen cell suspensions were obtained from the spleen by grinding the tissues directly and then passed through a sterile cell strainer with a pore size of 40 µm (BS-40-CS, Biosharp, China). The erythrocytes were lysed using RBC lysis buffer (R1010, Solarbio Biotech, China) for 5 min at 4°C. The resulting splenocytes were then suspended in RPMI 1640 medium, which was supplemented with 10% fetal calf serum (FCS). Cells were incubated with purified anti-mouse CD16/CD32 antibodies (E-AB-F0997A, Elabscience, China) to block Fc receptors on ice for 20 min. Then, cells were stained with immune-cell-specific antibodies for flow cytometry (Beckmann Coulter Inc., Fullerton, CA, USA) as follows: Alexa Fluor® 700 anti-CD3 antibody (100216, Biolegend, USA), FITC anti-CD4 antibody (100406, Biolegend, USA), Brilliant Violet 510™ anti-CD8a antibody (100752, Biolegend, USA) for CD3^+^ CD4^+^ or CD3^+^ CD8^+^ T cells. The frequency of IgG^+^ B_M_ cells was assessed on day 72 after the first immunization, and cells were stained with APC anti-IgG antibody (405308, Biolegend, USA), FITC anti-CD80 antibody (104706, Biolegend, USA), Alexa Fluor® 700 anti-IgD antibody (405730, Biolegend, USA), and Pacific Blue™ anti-CD45R/B220 antibody (103227, Biolegend, USA). The staining process was conducted on ice for 25 minutes. Then, cells were washed twice with 1 ml PBS (BL302A, Biosharp, China) and resuspended in 400 µl of staining buffer (PBS-1% BSA) for FACS analysis (Beckmann Coulter Inc., Fullerton, CA, USA). 7-AAD (420404, Biolegend, USA) was used to exclude dead cells. All data analysis was carried out using FlowJo software version 10.9.0 (FlowJo, FlowJo Ashland OR).

### Statistical analysis

The Graph-Pad Prism 8.0 software was utilized to conduct statistical calculations. Unless otherwise mentioned, numerical data were presented as means ± SEM. To assess differences in the levels of antibodies and the quantities of CD3^+^ CD4^+^ T cells, CD3^+^ CD8^+^ T cells, and IgG^+^ B_M_ cells, we conducted either one-way or two-way ANOVA analysis, subsequently applying Tukey’s multiple comparisons tests. The log-rank test was implemented to assess differences in mouse survival, with the Kaplan-Meier survival curve serving as the monitoring tool. To compare means, the least significant difference test was employed. A p-value below 0.05 was considered to indicate a meaningful difference.

## Results

### Construction of Salmonella mutants

**Figure 1.**
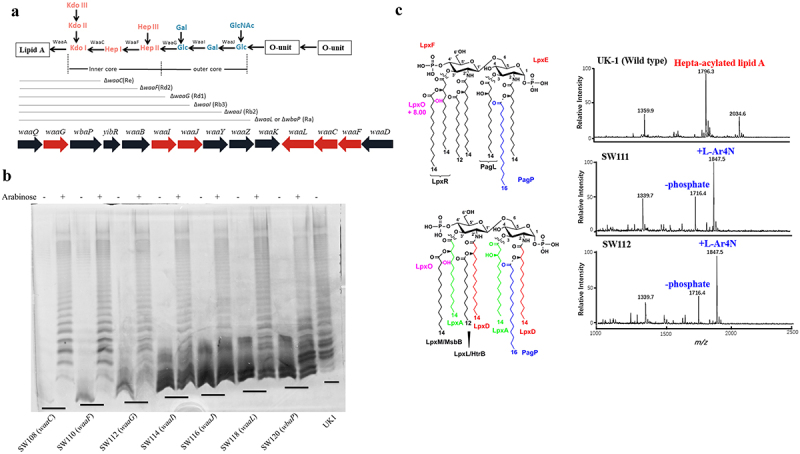
Construction of mutants and phenotypic determination. (a) all these mutants were derived from SW067 (Δ*pagL7* Δ*pagP81*::P_lpp_ *lpxE* Δ*lpxR9* Δ*fur9*). The genes *waaC*, *waaF*, *waaG*, *waaI*, *waaJ*, *waaL*, or *wbaP* were deleted from the genome of SW067, respectively. The gene *pagL* was replaced by TT *araC* P_BAD_ *waaC*, TT *araC* P_BAD_ *waaF*, TT *araC* P_BAD_ *waaG*, TT *araC* P_BAD_ *waaI*, TT *araC* P_BAD_ *waaJ*, TT *araC* P_BAD_ *waaL*, or TT *araC* P_BAD_ *wbaP* to generate a set of genetically modified strains capable of producing smooth LPS in response to arabinose regulation, respectively. (B) Upon the addition of arabinose in laboratory conditions, these mutants successfully synthesized a typical LPS pattern. In contrast, these mutants exhibited rough phenotypes in the absence of arabinose due to the loss of their ability the form LPS. (C) Lipid a structure of wild-type and *lpxE*-expressing SW111 (Δ*pagL7* Δ*pagP81*::P_lpp_ *lpxE* Δ*lpxR9* Δ*fur9* ∆*waaG42*) and SW112 (Δ*pagL76*::TT *araC* P_BAD_ *waaG* Δ*pagP81*::P_lpp_ *lpxE* Δ*lpxR9* Δ*fur9* ∆*waaG42*). In the negative-ion mode, using MALDI-TOF MS, one can detect the lipid a species. The unaltered lipid a is represented by the peak at *m/z* 1796.3.

To finely regulate the expression of genes related to LPS synthesis to control the synthesis of the native *Salmonella* LPS, we constructed at least two suicide plasmids carrying the TT *araC* P_BAD_ cassette and different SD sequences and/or start codons for each gene for LPS synthesis (Tables 1 and 2). These suicide plasmids were then utilized to integrate the TT *araC* P_BAD_ cassette carrying the candidate genes into the chromosome in place of the *pagL* gene since *pagL* deletion did not alter the virulence of *Salmonella* and maintained the same immunogenicity and colonization ability as the wild-type *Salmonella*.^25^ For an example of the regulated gene *waaC*, we first introduced the Δ*waaC* and Δ*pagL* mutation in the genome of χ3761 by allelic exchange via conjugation with the χ7213, harboring the plasmid of pSW012 or pYA4284, then two suicide plasmids (pSW033 and pSW034) were utilized to integrate the TT *araC* P_BAD_ cassette with *waaC* carrying two different SD sequences into the chromosome of SW086 (Δ*waaC40* Δ*pagL7*) in place of the *pagL* gene to generate mutants SW093 (Δ*waaC40* Δ*pagL71*::TT *araC* P_BAD_ *waaC*) and SW094 (Δ*waaC40* Δ*pagL72*::TT *araC* P_BAD_ *waaC*), respectively. The results of silver staining experiments showed that the mutant SW079 (Δ*waaC40*) was unable to synthesize a typical LPS banding pattern due to the deletion of the glycosyltransferase gene *waaC*, which is responsible for the addition of L-glycerol-D-mannoheptulose (Hep) residues to the core of LPS (Figure 1a, Supplementary Figure S1). When the mutants SW093 (Δ*waaC40* Δ*pagL71*::TT *araC* P_BAD_ *waaC*) and SW094 (Δ*waaC40* Δ*pagL72*::TT *araC* P_BAD_ *waaC*) were cultured *in vitro* without 0.1% arabinose, both SW093 (Δ*waaC40* Δ*pagL71*::TT *araC* P_BAD_ *waaC*) and SW094 (Δ*waaC40* Δ*pagL72*::TT *araC* P_BAD_ *waaC*) mutants failed to synthesize a typical LPS banding patterns, however only SW093 (Δ*waaC40* Δ*pagL71*::TT *araC* P_BAD_ *waaC*) was able to synthesize LPS banding pattern similar to the wild strain when arabinose was present (Supplementary Figure S1). This result suggested that the mutant SW093 (Δ*waaC40* Δ*pagL71*::TT *araC* P_BAD_ *waaC*) exhibited the ability to tightly regulate LPS synthesis. The same strategy was also applied to other genes, including *waaF, waaG*, *waaI*, *waaJ*, *waaL*, and *wbaP*. The integrations resulted in generating mutant strains with varying lengths of LPS (Figure 1a, Supplementary Figure S1).

After mutants with tightly regulated LPS were successfully identified in the wild-type background, these mutations were introduced into the mutant strain SW067 (χ9705 Δ*fur9*) to investigate whether the regulated LPS synthesis of *Salmonella* has an effect on the induced cross-immune response.

### Phenotype determination of the mutant strains

When the genes *waaC*, *waaF*, *waaG*, *waaI*, *waaJ*, *waaL*, or *wbaP* were deleted from the genome of SW067 (χ9705 Δ*fur9*), the mutant strains exhibited an inability to produce typical LPS banding pattern, indicating that the absence of any glycosyltransferase in LPS synthesis hinders the formation of a whole LPS (Supplementary Figure S2). When cultured in the presence of 0.1% arabinose, a series of the mutant strains with regulated LPS synthesis including SW108 (*waaC*), SW110 (*waaF*), SW112 (*waaG*), SW114 (*waaI*), SW116 (*waaJ*), SW118 (*waaL*), and SW120 (*wbaP*) exhibited typical LPS banding patterns while culturing in the absence of arabinose, these mutants displayed the rough LPS phenotype (Figure 1b). As the mutants will be administered orally and need to colonize the host organ, to evaluate whether they still display the tightly regulated LPS *in vivo*, these mutants were cultured *in vitro* in nutrient broth supplemented with varying concentrations of mouse serum, they were unable to synthesize the whole LPS in the absence of free arabinose (Supplementary Figure S3A). These results indicated that arabinose was unavailable in the serum and the synthesis of LPS in the mutants was entirely regulated by exogenously added arabinose. The OMPs profile showed differences in the expression levels of OMPs for the regulated attenuated *Salmonella* in this study, particularly for SW114 (*waaI*), SW116 (*waaJ*), SW118 (*waaL*), and SW120 (*wbaP*), indicating that the delayed attenuation system affects the expression of attenuated *Salmonella* OMPs (Supplementary Figure S3B). The MALDI-TOF MS analysis of lipid A showed that the phosphate group was removed to result in the production of monophosphorylated lipid A (Figure 1c).

### Colonization of the regulated delayed attenuated Salmonella

**Figure 2.**
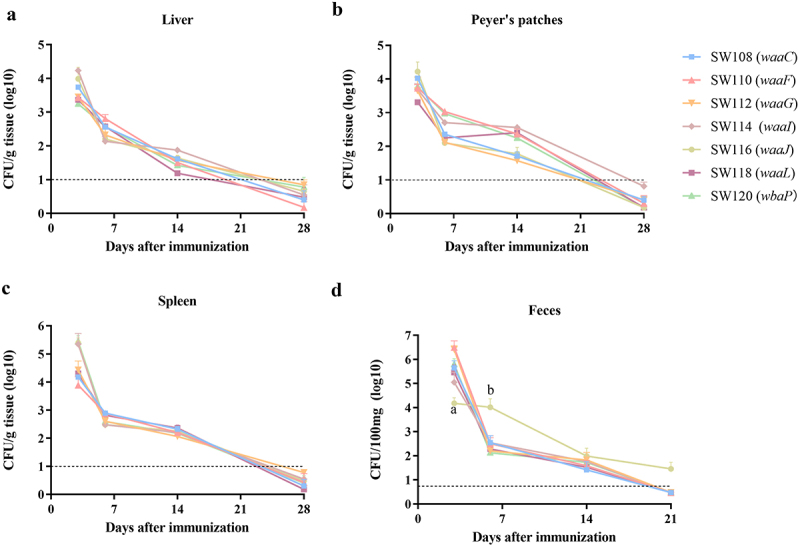
Bacterial burden in liver, spleen, Peryer’s patches, and feces of mice.Mice were given 20 µl of BSG including 1 × 10^9^ CFU mutants via oral route, and three mice from each group were humanely euthanized at 3, 6, 14, and 28d post-inoculation. The liver (a), spleen (b), and Peryer’s patches (c) were collected. After treating with tissue homogenizer, homogenized tissues were dropped on the LB and MacConkey agar after appropriate dilutions with phosphate buffer saline (PBS), and the bacterial loading was calculated based on the bacteria on the plates. (d) The bacterial load in feces. A total of 4-6 fecal pellets from immunized mice were collected in a 2-ml tube on days 3, 6, 14, and 21 following a single inoculation. Fecal pellets were weighed and homogenized in PBS (0.1 g/ml). The quantification of bacteria per 100 mg of feces was performed by dropping serially diluted or undiluted fecal homogenate on an XLD agar plate. The standard differences between the mice in each group were shown by the error bars. The dotted black line indicates the minimal detection limit of the test, which is 10 CFU/g or 5 CFU/100 mg. Data are presented as the means ± SEM (*n* = 3). Superscript letters a and b indicate *p* < 0.05 for comparisons with the SW118 (*waaL*) group on days 3 and 6 following a single inoculation, respectively.

The colonization levels in the organs of mice reflect the interaction between the attenuated *Salmonella* and the lymphoid tissue, impacting the capacity of attenuated *Salmonella* to elicit immune responses. To investigate the dissemination and proliferation capabilities of the regulated delayed attenuated *Salmonella* in mice, we assessed the bacterial burdens in the liver, spleen, and Peyer’s patches of mice at 3, 6, 14, and 28 days following oral administration with 1 × 10^9^ CFU/20 µl of the mutant strains, which were grown in the presence of 0.1% arabinose. The average CFU counts of all mutant strains isolated from the liver, Peyer’s patches, or spleen were approximately 10^4^ at 3 days after oral inoculation (Figure 2a-c), indicating that attenuated *Salmonella* maintained the ability to invade and colonize the gastrointestinal tract and were rapidly disseminated to systemic sites and underwent substantial proliferation within the organs. At 6 days post-infection, all mutant strains exhibited decreased colonization levels in the liver, Peyer’s patches, and spleen. All mutant strains colonized and persisted in the organs for at least 28 days, and did not show significant differences. Within these 28 days, the bacterial counts in the liver, Peyer’s patches, and spleen gradually decreased, suggesting that the regulated delayed attenuated *Salmonella* could be cleared after colonization, and the abnormal appearance of the skin and other symptoms were not observed during the current experiment. We also investigated the levels of *Salmonella* in fecal shedding in mice vaccinated with the regulated delayed attenuated *Salmonella* in this study. The results showed that at least 10^4^ CFU/100 mg of *Salmonella* were detected in the fecal shedding of mice inoculated with the regulated delayed attenuated *Salmonella* at day 3, and a small number of *Salmonella* remained present on day 14 (Figure 2d). On day 21, the amount of *Salmonella* in fecal shedding was below the level where it could be detected in all but the SW116 (*waaJ*) group of mice (Figure 2d).

To investigate the effect of arabinose in the intestine on the colonization of regulated delayed attenuated *Salmonella*, arabinose was added to the drinking water of the mice inoculated with SW108 (*waaC*) or SW118 (*waaL*), and we evaluated the bacterial loads in the spleen, liver, and Peyer′s patches of mice on days 3, 6, 14, and 21 after the initial immunization. Throughout the experiment, the supplementation or non-supplementation of arabinose in the drinking water of immunized mice had no significant effect on the level of SW108 (*waaC*) colonization (Supplementary Figure S4). For SW118 (*waaL*), the number of bacteria in the spleens of mice with arabinose added to the drinking water was significantly higher than in mice without arabinose added to the drinking water on day 3 after the first immunization, however, there was no statistically significant difference in the number of bacteria in the liver and Peyer’s patches (Supplementary Figure S4B). The bacterial burden in the spleen, liver, and Peyer′s patches of the mice in each experimental group on days 6, 14, and 21 following the initial immunization did not significantly differ (Supplementary Figure S4).

### Histological analysis after immunization with the mutant strains

**Figure 3.**
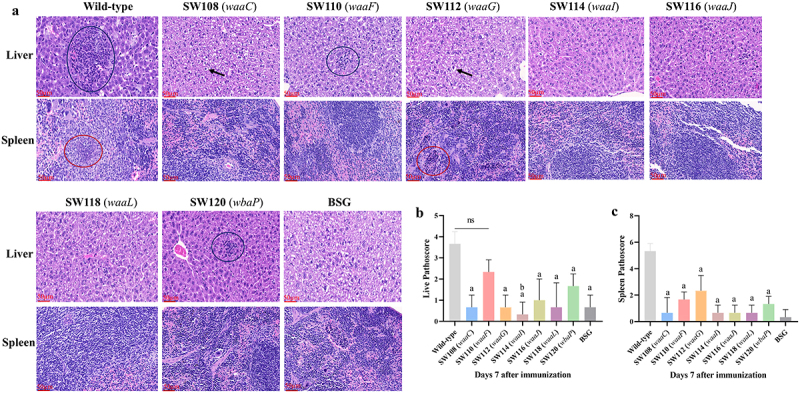
Histopathological analysis.Analysis.The effects of regulated delayed attenuated *Salmonella* on the liver and spleen were assessed by histopathological examination at 7 days post-inoculation. (a) Representative histopathological photographs of the liver and spleen of immunized mice. The black arrow symbolizes hepatocytes. The blue circles depicted in the image signify areas of focal necrosis. The red circles depict the infiltration of neutrophils. Images were captured with a magnification of 10 × (scale bars 250 μm) and an inset magnification of 40 × (scale bars 50 μm). Enumeration of liver (b) and spleen (c) pathology in terms of an 8-point or 12-point pathoscore scale was assessed as described above (see the material and methods section), respectively. The standard differences between the mice in each group were shown by the error bars. Data are presented as the means ± SEM (*n* = 3). Superscript letters a and b indicate *p* < 0.05 for comparisons with the BSG and SW110 (*waaF*) groups, respectively.

To investigate the potential of the regulated delayed attenuated *Salmonella* to induce tissue inflammation, we conducted histopathology analyses on the liver and spleen of mice inoculated with mutant strains, using BSG and wild-type *S*. Typhimurium (χ3761) as the control. The mice were orally inoculated with 1 × 10^9^ CFU of the mutant strains or wild-type *S*. Typhimurium (χ3761) in the 20 µl BSG, which were cultured in LB with 0.1% arabinose, and the examinations were performed 7 days post-infection. The results showed that the pathoscores of the liver tissue of mice belonging to the SW108 (*waaC*), SW112 (*waaG*), SW114 (*waaI*), SW116 (*waaJ*), SW118 (*waaL*), and SW120 (*wbaP*) groups were significantly lower than those of the wild-type control group, except for SW110 (*waaF*) group (Figures 3a,b). The SW110 (*waaF*) and SW120 (*wbaP*) groups showed higher pathoscores than the SW108 (*waaC*), SW112 (*waaG*), SW116 (*waaJ*), and SW118 (*waaL*) groups, indicating the SW110 (*waaF*) and SW120 (*wbaP*) remained virulent (Figures 3a,b). The pathoscores of the spleen tissue in the SW112 (*waaG*) group were comparatively greater than those of the other experimental groups, while statistical analysis did not find a difference (Figure 3c). No noticeable lesions were observed in mice from the SW108 (*waaC*), SW114 (*waaI*), SW116 (*waaJ*), and SW118 (*waaL*) groups. These results implied that the SW108 (*waaC*), SW114 (*waaI*), SW116 (*waaJ*), and SW118 (*waaL*) were avirulent in mice.

### Serum IgG and mucosal IgA responses to OMPs from S. Typhimurium

**Figure 4.**
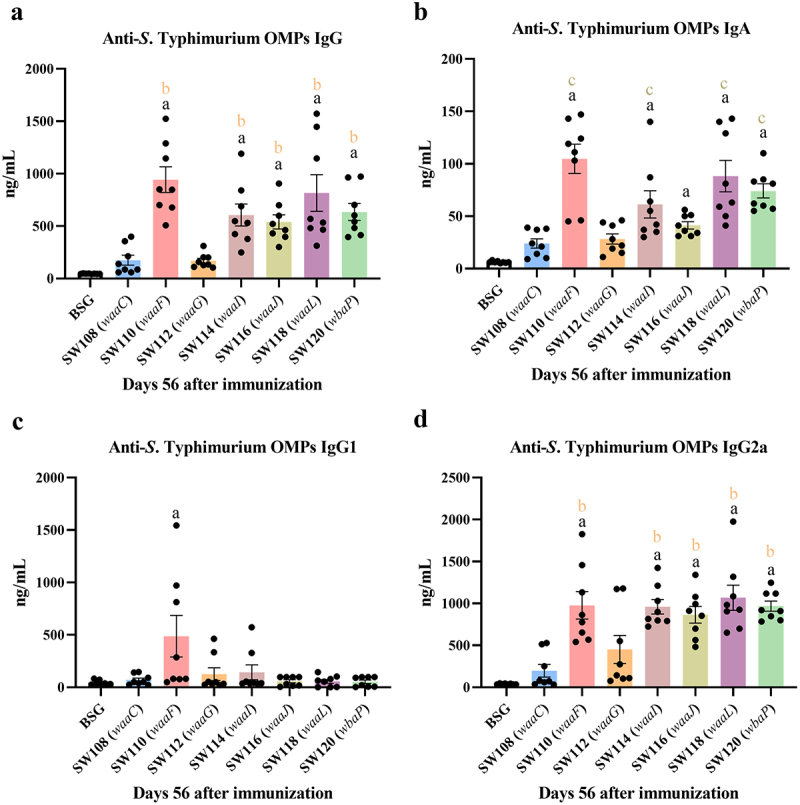
Immune response to OMPs from *S*. Typhimurium induced by the regulated delayed attenuated *Salmonella*.Serum and vaginal secretions of mice were gathered at week 8 post-initial vaccination. Quantitative ELISA was applied to analyze the levels of specific IgG (a), IgA (b), IgG1 (c), and IgG2a (d) against *S*. Typhimurium OMPs. The results displayed the precise levels of antibodies, as measured by a standard curve, in mice orally inoculated with the regulated delayed attenuated *Salmonella* at the scheduled weeks. The standard differences between the mice in each group were shown by the error bars. Data are presented as the means ± SEM (*n* = 8), superscript letters a, b, and c indicate *p* < 0.05 for comparisons with the BSG, SW112 (*waaG*), and SW116 (*waaJ*) groups, respectively.

To evaluate the immune responses triggered by the regulated delayed attenuated *Salmonella*, a total of 8 sets of mice were administered with 20 µl of BSG consisting of 1 × 10^9^ CFU strains via the oral route. Subsequently, these mice were boosted with an identical dose of the matching mutants 4 weeks later. The levels of serum IgG and mucosal IgA responses toward OMPs derived from *S*. Typhimurium were assessed using the ELISA method at 8 weeks after initial immunization. The SW114 (*waaI*), SW116 (*waaJ*), SW118 (*waaL*), and SW120 (*wbaP*) strains elicited considerably increased levels of IgG and IgA specific to *S*. Typhimurium OMPs compared to the SW108 (*waaC*), SW112 (*waaG*) and BSG groups (Figure 4a,b). Moreover, the SW110 (*waaF*) strain, which synthesized native *S*. Typhimurium LPS in the presence of arabinose *in vitro* and produced only a partial inner core of LPS in the absence of arabinose *in vivo*, also triggered mice to produce similar levels of *S*. Typhimurium OMPs-specific IgG and IgA antibodies as the SW118 (*waaL*) and SW120 (*wbaP*) strains, which both synthesized an intact inner and outer core of LPS in the absence of arabinose *in vivo* (Figure 4a,b). Although the levels of IgA specific to *S*. Typhimurium OMP triggered by the SW116 (*waaJ*) strain were significantly enhanced compared to the BSG control groups, it was lower than that of the SW110 (*waaF*), SW114 (*waaI*), SW118 (*waaL*), and SW120 (*wbaP*) immunized groups (Figure 4b). We also assessed the levels of anti-OMP IgG isotype subclasses IgG1 and IgG2a in the serum of mice. At the fourth week after the first immunization, the IgG1 titers were comparable to the levels of IgG2a (data not presented). However, the SW114 (*waaI*), SW116 (*waaJ*), SW118 (*waaL*), and SW120 (*wbaP*) strains stimulated a more rapid increase in IgG2a titers than IgG1 titers compared to the SW108 (*waaC*), SW112 (*waaG*) and BSG control groups at the eighth week after the initial immunization, suggesting a shift toward a Th1-biased immune response (Figure 4c,d). Notably, the SW110 (*waaF*) strain not only provoked significantly increased IgG2a but also elicited *S*. Typhimurium OMP-specific subclasses IgG1.

### Evaluation of cross-reactive antibodies against OMPs from other Salmonella serotypes

**Figure 5.**
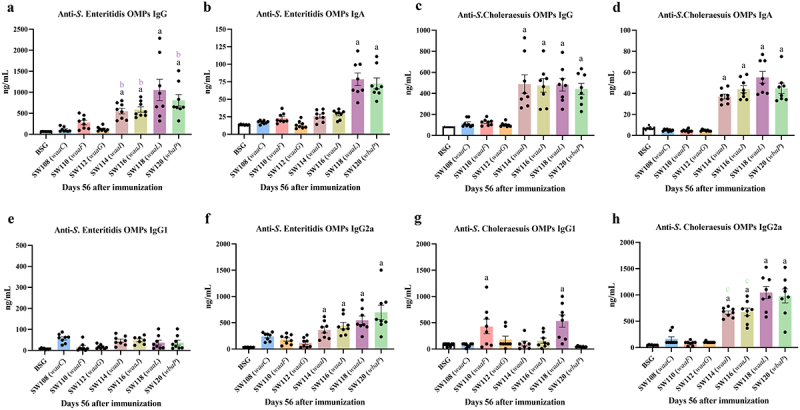
Immune response to OMPs from *S*. Choleraesuis and *S*. Enteritidis induced by the regulated delayed attenuated *Salmonella*.Serum and vaginal secretions of mice were gathered at week 8 post-initial vaccination. Quantitative ELISA was applied to analyze the levels of specific IgG (a, c) and IgA (b, d) against OMPs from *S*. Enteritidis and *S*. Choleraesuis, as well as the subtypes IgG1 (e, g) and IgG2a (f, h). The results displayed the precise levels of antibodies, as measured by a standard curve, in mice orally inoculated with the regulated delayed attenuated *Salmonella* at the scheduled weeks. The standard differences between the mice in each group were shown by the error bars. Data are presented as the means ± SEM (*n* = 8), superscript letters a, b, and c indicate *p* < 0.05 for comparisons with the BSG, SW114 (*waaI*), and SW120 (*wbaP*) groups, respectively.

To further analyze the cross-immune response induced by the regulated delayed attenuated *Salmonella* in mice, we assessed serum IgG and mucosal IgA responses to *S*. Enteritidis and *S*. Choleraesuis OMPs in mice at 8 weeks after the initial immunization and evaluated protection against the challenges of wild type *S*. Enteritidis and *S*. Choleraesuis at 9 weeks after the initial immunization. Not only did the SW118 (*waaL*) and SW120 (*wbaP*) mutants stimulate *S*. Enteritidis OMPs-specific IgG production, but also the SW114 (*waaI*) and SW116 (*waaJ*) mutants elicit higher levels of IgG specific to *S*. Enteritidis OMP than the control groups of BSG (Figure 5a). The SW118 (*waaL*) mutant, but not SW120 (*wbaP*), could elicit the highest *S*. Enteritidis OMP-specific IgG titer among all groups. The SW118 (*waaL*) and SW120 (*wbaP*) strains induced the production of *S*. Enteritidis OMP-specific vaginal IgA, whereas the SW114 (*waaI*) and SW116 (*waaJ*) strains failed to elicit vaginal mucosal IgA responses (Figure 5b). The levels of *S*. Choleraesuis OMP-specific serum IgG and mucosal IgA triggered by the SW114 (*waaI*), SW116 (*waaJ*), SW118 (*waaL*), and SW120 (*wbaP*) strains were significantly enhanced compared to those observed in the other treatment groups (Figure 5c,d).

Further analysis indicated that IgG2a was the major subtype of OMP-specific IgG triggered by the regulated delayed attenuated *Salmonella* (Figure 5e-h). The SW114 (*waaI*), SW116 (*waaJ*), SW118 (*waaL*), and SW120 (*wbaP*) strains could stimulate similar levels of *S*. Enteritidis OMP-specific IgG2a compared to SW108 (*waaC*), SW110 (*waaF*) and SW112 (*waaG*) strains (Figure 5f). Interestingly, in addition to the SW110 (*waaF*) strains triggered *S*. Choleraesuis OMP-specific IgG1 production, the SW118 (*waaL*) mutants, which synthesize both the complete inner and outer cores of the LPS in the absence of arabinose *in vivo*, also induced similar levels of *S*. Choleraesuis OMP-specific IgG1 (Figure 5g). Both the SW114 (*waaI*) and SW116 (*waaJ*) strains induced significantly enhanced levels of *S*. Choleraesuis OMP-specific IgG2a relative to the SW108 (*waaC*), SW110 (*waaF*), SW112 (*waaG*) strains, but lower than the SW118 (*waaL*) and SW120 (*wbaP*) strains (Figure 5h).

### Serum antibody-mediated complement deposition

**Figure 6.**
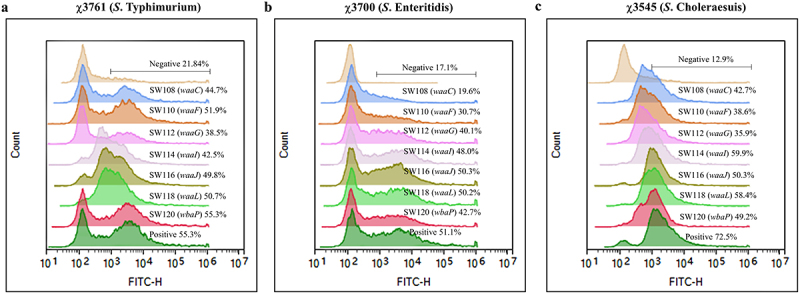
Complement deposition assays.Serum samples from the same group of mice previously collected at the 4th week after the second immunization were mixed and heat-inactivated. A 1 × 10^7^ CFU/100 µl wild-type *S*. Typhimurium (χ3761) (a), *S*. Enteritidis (χ3700) (b) or *S*. Choleraesuis (χ3545) (c) strain was resuspended in 80 µl of PBS with 1% BSA and mixed with 20 µl of heat-inactivated pooled serum from the relevant group. The mixtures were incubated at 37°C for 40 minutes. Subsequently, the treated *Salmonella* cells were washed with PBS and exposed to 100 µl of PBS-1% BSA solution containing rabbit complement and incubated at 37°C for 30 minutes. The treated *Salmonella* strain was stained with fitc-conjugated goat anti-rabbit complement polyclonal antibody and examined using flow cytometry. Positive control sera are polyclonal antibodies against *Salmonella* O4, O7, or O9 O-antigen, and sera for negative controls were obtained from mice oral with BSG. The data presented represents one experiment out of two. The numbers on the graph indicate the percentage of C3 complement deposition.

Serum antibody-mediated complement deposition is a crucial mechanism by which the host immune system defends itself against pathogens and regulates the immune response, and is considered a potential *in vitro* indicator of protection against many pathogenic microorganisms.^42^ The serum antibody-mediated complement deposition for wild-type *S*. Typhimurium, *S*. Enteritidis, or *S*. Choleraesuis using pooled sera from vaccinated mice was conducted to assess the functionality of serum antibodies elicited by the regulated delayed attenuated *Salmonella* in mice (Figure 6). C3 was only a trace amount deposited when sera from BSG-treated immunized mice were incubated with wild-type *S*. Typhimurium, *S*. Enteritidis, or *S*. Choleraesuis. On the contrary, sera from SW114 (*waaI*), SW116 (*waaJ*), SW118 (*waaL*), and SW120 (*wbaP*) groups resulted in high levels of C3 deposition on wild-type *S*. Typhimurium, *S*. Enteritidis, or *S*. Choleraesuis. Sera from the SW108 (*waaC*) immunized mice could also deposit C3 on the surface of wild-type *S*. Typhimurium and *S*. Choleraesuis compared to BSG control, but not on the *S*. Enteritidis. C3 deposition was also observed when we used sera from the SW112 (*waaG*) or SW110 (*waaF*) immunized group (Figure 6a-c).

### Determination of T-cell responses

**Figure 7.**
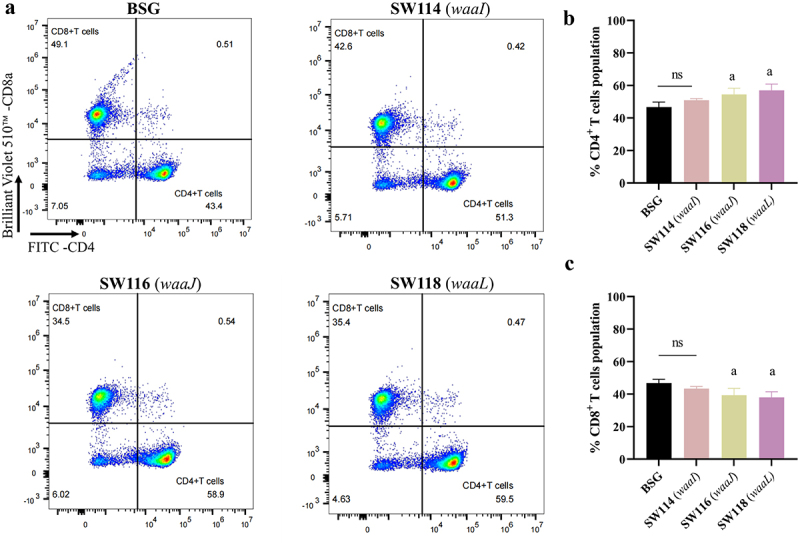
Determination of T-cell responses.T-cell responses were investigated by flow cytometric analysis of the spleen for T-cell markers CD3^+^ CD4^+^ and CD3^+^ CD8^+^ at 38 days after the initial immunization (10 days after the second vaccination). The proportions of CD3^+^ CD4^+^ and CD3^+^ CD8^+^ T cells were determined by gating. (A) The representative dot plots derived from the flow cytometry analysis were illustrated in the figure, with the indicated gating and cell populations. The percentage of CD3^+^ CD4^+^(B) and CD3^+^ CD8^+^(C) T cells was calculated in three samples. The standard differences between the mice in each group were shown by the error bars. Data are presented as the means ± SEM (*n* = 3), superscript letters a indicate *p* < 0.05 for comparisons with the BSG group, while ns indicates no significant difference.

T-cell responses were investigated by flow cytometric analysis of the spleen for T-cell markers CD3^+^ CD4^+^ and CD3^+^ CD8^+^. As SW114 (*waaI*), SW116 (*waaJ*), and SW118 (*waaL*) performed well in inducing antibody production, they were chosen to assess T-cell responses. The spleen samples were collected from immunized mice 38 days after the initial immunization (10 days after the second vaccination). The proportions of CD3^+^ CD4^+^ and CD3^+^ CD8^+^ T cells were determined by gating (Supplementary Figure S5). The results showed a significant increase in the maturation of CD4^+^ markers in the SW113 (*waaJ*) and SW116 (*waaL*) groups compared to the SW110 (*waaI*) and BSG control groups, and the proportion of CD4^+^ T cells was greater than that of CD8^+^ T cells, indicating a preference for CD4^+^ responses over CD8^+^ responses (Figure 7a-c Supplementary Figure S6).

### Assessment of long-term immune responses induced by regulated delayed attenuated Salmonella

A successful vaccine must effectively elicit and establish lasting memory immunity; therefore, we investigated the long-term immune responses triggered in mice following regulated delayed attenuated *Salmonella* immunization via oral administration. As SW114 (*waaI*), SW116 (*waaJ*), and SW118 (*waaL*) performed well in inducing antibody production, they were chosen to explore their abilities to induce long-term immunity. The IgG antibody levels in the serum of immunized mice were quantified on days 72 and 120 following the initial immunization. The results showed that *S*. Typhimurium, *S*. Enteritidis, and *S*. Choleraesuis OMP-specific IgG were still observable in all experimental groups compared to the BSG control group on 72 and 120 days (Supplementary Figure S7). The levels of IgG antibodies in the SW118 (*waaL*) group exhibited a statistically significant increase compared to both the SW114 (*waaI*) and SW116 (*waaJ*) groups.

**Figure 8.**
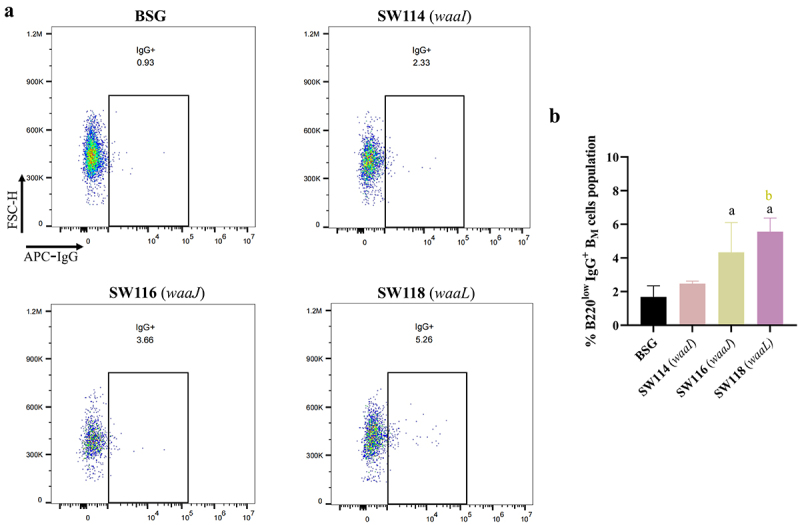
The assessment of long-term immune responses induced by regulated delayed attenuated *Salmonella*.The percentage of the B220^low/int^ CD80^+^ IgD^−^ IgG^+^ B_M_ cells from immunized mice was evaluated by flow cytometry on day 72 after the initial immunization. (A) The representative dot plots derived from the flow cytometry analysis were illustrated in the figure, with the indicated gating and cell populations. (B) The percentage of B220^low^ CD80^+^ IgD^−^ IgG^+^ B_M_ cells was calculated in three samples. The standard differences between the mice in each group were shown by the error bars. Data are presented as the means ± SEM (*n* = 3), superscript letters a and b indicate *p* < 0.05 for comparisons with the BSG and SW116 (*waaJ*) groups, respectively.

The establishment of a durable population of memory B cells is crucial for a vaccine to develop sustained immunity.^43^ In this study, we gated B220^low/int^ CD80^+^ IgD^−^ IgG^+^ B_M_ cells to evaluate IgG^+^ B_M_ cells by conducting flow cytometry analysis on day 72 after the first immunization (Supplementary Figure S8).^32,44,45^ The percentage of B220^low^ CD80^+^ IgD^−^ IgG^+^ B_M_ cells in the groups immunized with the SW116 (*waaJ*) was considerably increased compared to the SW114 (*waaI*) and BSG groups but less than the SW118 (*waaL*) immunized group (Figure 8a,b Supplementary Figure S9). However, there was no significant difference in the percentage of B220^high^ CD80^+^ IgD^−^ IgG^+^ B_M_ cells between all experimental groups and the BSG group (data not presented). These findings indicated that the regulated delayed attenuated *Salmonella* constructed in this study, particularly the SW118 (*waaL*), which both synthesized an intact inner and outer core of LPS in the absence of arabinose *in vivo*, could elicit and establish lasting memory immunity in mice.

### Evaluation of protection against challenge by wild-type S. Typhimurium

**Figure 9.**
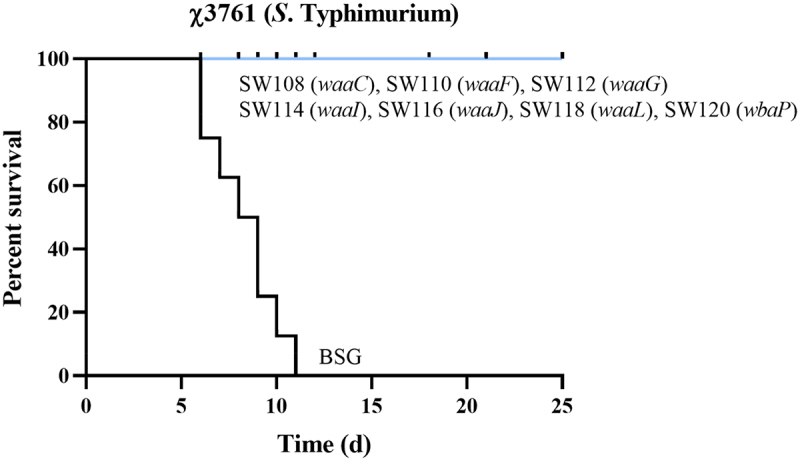
Evaluation of protection against challenge by wild-type *S*. Typhimurium.Typhimurium.The immunized mice underwent an oral challenge with 10^9^ CFU (10000 × LD_50_) of χ3761 in 20 µl BSG at week 9 after the initial immunization (*n* = 8). Following the challenge, mortality was recorded for 25 days.

To investigate the protection rates conferred by the regulated delayed attenuated *Salmonella*, the immunized mice were orally challenged with 1 × 10^9^ CFU (10000 × LD_50_) of χ3761 in 20 µl BSG at 9 weeks after the first immunization. The results showed that all of the mice in the BSG control group died; In contrast, all the vaccinated mice survived (Figure 9), implying that all of the regulated delayed attenuated *Salmonella* were able to provide complete protection to the mice against the wild-type *S*. Typhimurium challenge.

### Evaluation of cross-protection against infection with heterologous serotype of Salmonella

**Figure 10.**
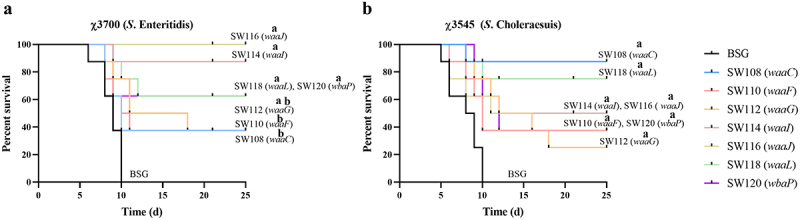
Evaluation of cross-protection against challenge by *S*. Enteritidis or *S*. Choleraesuis.Choleraesuis.The immunized mice underwent an oral challenge with 10^7^ CFU (100 × LD_50_) of χ3700 (A), or 10^7^ CFU (100 × LD_50_) of χ3545 (b) in 20 µl BSG at week 9 after the initial immunization (*n* = 8), respectively. Following the challenge, mortality was recorded for 25 days. The superscript letters a and b indicate *p* < 0.05 for comparisons with the BSG and SW116 (*waaJ*) groups, respectively.

To evaluate protection against the challenges of different serotypes of *Salmonella*, sixteen groups of BALB/c mice were used to immunize, including fourteen experimental groups and two control groups. The mice in each group were independently challenged by oral administration with 1 × 10^7^ CFU (~100 × LD_50_) of χ3700 (*S*. Enteritidis) or χ3545 (*S*. Choleraesuis) in 20 µl BSG, respectively. As shown in Figure 10a, the SW116 (*waaJ*), SW114 (*waaI*), SW118 (*waaL*), SW120 (*wbaP*), and SW112 (*waaG*) provided significant protection against wild-type *S*. Enteritidis challenge compared to the BSG controls. In comparison to the SW118 (*waaL*) and SW120 (*wbaP*), immunizations with the SW116 (*waaJ*) and SW114 (*waaI*) vaccines indicated somewhat higher levels of protection; however, this trend was not of statistical significance. The SW116 (*waaJ*) conferred a prominent level of protection against wild-type *S*. Enteritidis challenge in contrast to the SW112 (*waaG*), SW110 (*waaF*), and SW108 (*waaC*).

The protection rate against the challenge of *S*. Choleraesuis was inconsistent with the level of protection against *S*. Enteritidis. All regulated delayed attenuated *Salmonella* provided significant protection against wild-type *S*. Choleraesuis (Figure 10b). The SW118 (*waaL*), SW114 (*waaI*), and SW116 (*waaJ*) mutants conferred protection rates of 75% and 50% for mice against the wild-type *S*. Choleraesuis challenge, respectively (Figure 10b). Interestingly, although SW108 (*waaC*) did not confer a high level of protection against the *S*. Enteritidis challenge, it provided the highest level of protection against the *S*. Choleraesuis challenge with a survival rate of 87.5% (Figure 10b).

### Evaluation of immunogenicity against OMPs from different enteric bacteria

**Figure 11.**
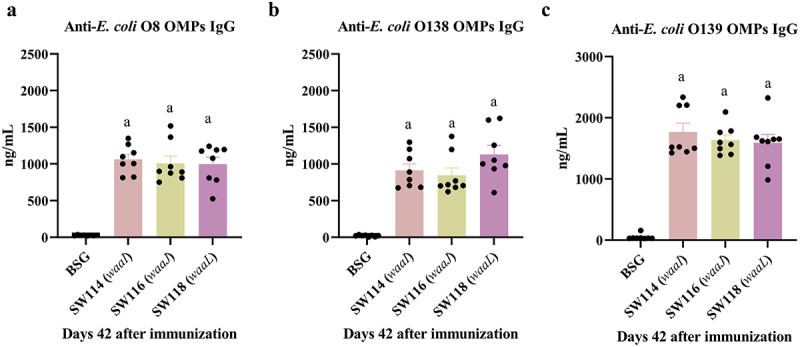
Immune response to OMPs from other enteric bacteria induced by the regulated delayed attenuated *Salmonella*.The sera were gathered from mice in SW114 (*waaI*), SW116 (*waaJ*), and SW118 (*waaL*) groups on days 42 after the primary immunization. Quantitative ELISA was applied to analyze the levels of specific IgG against OMPs from *E. coli* O8 (A), *E. coli* O138 (B), and *E. coli* O139 strain (C). The results displayed the precise levels of antibodies, as measured by a standard curve. The standard differences between the mice in each group were shown by the error bars. Data are presented as the means ± SEM (*n* = 8), superscript letters a indicate *p* < 0.05 for comparisons with the BSG groups.

Our previous research has demonstrated that the absence of O-antigen stimulates elevated levels of antibodies against the outer membrane protein antigen in serum, and these antibodies exhibit cross-reactivity with conserved surface epitopes present in other enteric bacteria.^19,20,26^ Therefore, we determined whether the regulated delayed attenuated *Salmonella* mutants had a similar effect. The reactivity of immune sera obtained from orally immunized mice belonging to SW114 (*waaI*), SW116 (*waaJ*), and SW118 (*waaL*), as described in the previous experiment (Figure 5), was evaluated using ELISA against OMPs isolated from several wild-type *E. coli* (O8, O138, O139) strains (Figure 11). The results roughly correlated with that we obtained against OMPs of heterologous serotype *Salmonella* (Figure 5). The three vaccine strains elicited a significant reactivity response in mice against OMPs from several *E. coli* strains compared to the BSG control group, and there was no statistical difference between the groups (Figure 11).

## Discussion

This study investigated the immunogenicity and protective efficacy of the regulated delayed attenuated *Salmonella* mutants in mice. The goal was to develop potentially regulated delayed attenuated *Salmonella* that could provide protection against both homologous and heterologous serotype *Salmonella* infections. The serotypes of *S*. Typhimurium, *S*. Enteritidis, and *S*. Choleraesuis in the NTS^46^ belong to serogroups B1, D1, and C1, respectively, and have many natural hosts such as mice, chickens, poultry, and pigs.^47,48^ Numerous studies have demonstrated that these serotypes have the ability to establish systemic infections in mice after oral administration.^17,49–52^ Therefore, the mouse model was chosen to evaluate the immunogenicity of the regulated delayed attenuated *Salmonella* mutants and their cross-protection efficiency against *S*. Typhimurium, *S*. Choleraesuis, and *S*. Enteritidis infections in this study. These mutant strains were derived from the same genetic background, thus avoiding any potential interference factors to compare immunogenicity and cross-protection efficiency induced by these strains.

**Figure 12.**
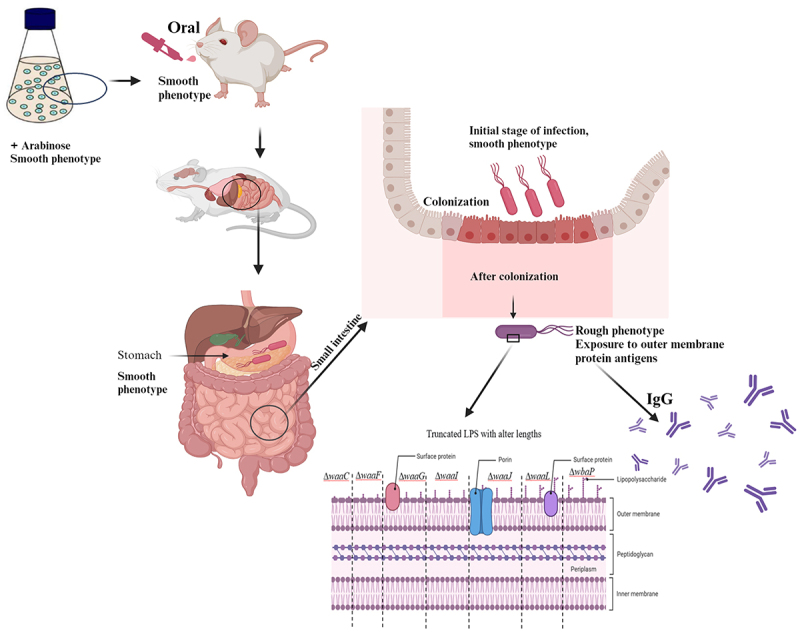
The characteristics of the regulated delayed attenuated *Salmonella*.The regulated delayed attenuated *Salmonella* are capable of synthesizing full LPS lengths by utilizing arabinose and maintaining smooth LPS patterns to keep their original ability to colonize the small intestines at the initial stage of the infection after immunization of mice via the oral route. Subsequently, different truncated lengths of LPS were synthesized in the absence of arabinose *in vivo*, thereby exposing conserved protein antigens on the outer membrane for inducing the production of specific IgG antibodies.

Successful colonization of attenuated *Salmonella* is a prerequisite for inducing an effective immune response in mice, and the intact LPS structure is essential for *Salmonella* adhesion and colonization in the intestine and systemic infection.^26^ Previous studies have reported that while permanent deficiency of the O-antigen reduces host organ damage by *Salmonella*, it also affects the level of colonization in mice.^18,53^ In this study, a series of *Salmonella* mutants with arabinose-dependent regulated synthesis of LPS were constructed, and the results have shown that at least one of them carrying altered length of LPS was successfully identified to be tightly regulated by arabinose in LB media and Nutrient broth with mice serum (Figure 1b), which was consistent with our previous studies.^18,32^ The regulated delayed attenuated *Salmonella* constructed in this study can control the synthesis of *Salmonella* LPS by adding arabinose to the culture medium, so that *Salmonella* still has an intact LPS structure at the early stage of colonization in mice (Figure 12).

The regulated delayed attenuated *Salmonella* exhibited a persistent ability of colonization in the initial stages of infection, at least maintaining this level for 6 days (Figure 2a-c), indicating that these mutants with regulated LPS synthesis were able to successfully colonize the liver, spleen, and peyer’s patches of mice due to the presence of the intact LPS, which is consistent with the timeframe required for antigen recognition and lymphocyte activation leading to the development of adaptive immunity. Subsequently, the level of colonization of the mutants gradually decreased in mice, suggesting that the mutants could be cleared after inducing immunity, thus ensuring the safety of candidate vaccines. The shedding of *Salmonella* in feces can facilitate the spread of *Salmonella* to other hosts by contaminating water and food sources and is an important route for the transmission of *Salmonella* between hosts. Although our data showed that mice immunized with regulated delayed attenuated *Salmonella* continued to shed *Salmonella* from feces for at least 14 days (Figure 2d), the chances of transmission of secondary infections via immunized mice were low because host-to-host transmission needs to be a significant amount of *Salmonella* present in the feces and the regulated delayed attenuated *Salmonella* was weak and more sensitive to the adverse factors in the environment.^54,55^

Live attenuated *Salmonella* vaccines must balance safety with immunogenicity,^56^ which can be achieved by incorporating a minimum of two genetically unlinked attenuating mutations. Hence, we constructed *Salmonella* mutants with the *fur* mutation and the arabinose-regulated LPS synthesis coupled with the deletion of *pagL*, *lpxR*, and codon-optimized *lpxE* substitution for *pagP* to engineer *Salmonella* synthesizing the 4′-monophosphoryl-hexa-acylated lipid A, which decreases the interaction of lipid A and TLR4 and endotoxic activity while maintaining its good immunogenicity.^25^ Histopathological assay conducted on mice 7 days post-immunization demonstrated that delayed attenuated *Salmonella* mutants exhibited varying degrees of inflammation. Mice that were immunized with the strains SW108 (*waaC*) and SW110 (*waaF*), which are characterized by the deep rough phenotype and have a partial inner core of LPS in the absence of arabinose *in vivo*, only SW110 (*waaF*) showed localized damage to liver cells. Although the mutants SW112 (*waaG*), SW114 (*waaI*), and SW116 (*waaJ*), which had an intact inner core or partial outer core of LPS in the absence of arabinose *in vivo*, just strain SW112 (*waaG*) caused damage to spleen tissue (Figure 3). Interestingly, no obvious abnormalities were observed in mice infected with strains SW114 (*waaI*) and SW116 (*waaJ*). While the strains SW118 (*waaL*) and SW120 (*wbaP*) had both the inner and outer core of LPS but lacked the O-antigen in the absence of arabinose *in vivo*, mice that were immunized with strain SW118 (*waaL*) did not exhibit any observable abnormalities, while those inoculated with strain SW120 (*wbaP*) showed small areas of necrosis in the liver. These results indicated that mutants exhibiting deep rough phenotype may still have significant adverse effects due to slower generation of a rough phenotype *in vivo*. Additionally, the process of LPS synthesis is intricate and subtle, even when the degree of truncated LPS is identical, the resulting properties observed in mice may differ.

Attenuated live *Salmonella* vaccines have the ability to induce antibodies against conserved outer membrane proteins that are cross-reactive with other enteric pathogens, particularly in strains engineered to achieve down-regulation of O-antigen synthesis *in vivo*. The immune responses observed in mice following immunization with regulated delayed attenuated *Salmonella* were found to be varied. Despite the absence of a significant antibody response against OMPs of *S*. Typhimurium when mice were administered SW108 (*waaC*) and SW112 (*waaG*), these mutants still provided complete protection in mice when exposed to a lethal dose of highly virulent wild-type *S*. Typhimurium (Figures 4, 9). Considering that the regulated delayed attenuated *Salmonella* originated from UK1, this result is not unexpected, and it can be attributed to the presence of various surface antigens in *S*. Typhimurium, such as flagellins, fimbriae, and enterobacterial common antigens (ECA), and the removal of immunodominant O-antigens from the surface of *Salmonella* also enhances the immunogenicity of OMPs and other surface antigens.

Our aim was to develop a live attenuated *Salmonella* to induce cross-immune responses effectively and confer cross-protection against infection of multiple serotypes of *Salmonella*. In this study, mice in groups SW116 (*waaJ*), SW114 (*waaI*), and SW118 (*waaL*) exhibited significant antibody levels and a considerable survival rate when challenged with both wild-type *S*. Enteritidis and *S*. Choleraesuis, but mice in the SW108 (*waaC*) group, which exhibited negligible antibodies against OMPs from *S*. Choleraesuis, had the highest survival rate compared to the other groups (Figures 5, 10). In addition, mice in group SW120 (*wbaP*), which displayed the identical LPS as SW118 (*waaL*), were characterized by elevated levels of antibodies, but displayed a considerably lower survival rate of only 37.5%, implying that no apparent relationship exists between antibody levels against OMPs assessed in mice immunized with mutants and protection when challenged with other *Salmonella*. It seems that the conclusion made in this study was not consistent with other research.^57^ *Salmonella* possesses many immunologically related cross-reactive OMP antigens, including abundant (OmpA, OmpC, and OmpD) and minor (e.g., NmpC and OmpX) proteins as well as the most copious lipoprotein (murein lipoprotein). These OMPs, although possessing some micro-heterogeneity, nevertheless share antigenic determinants, which can induce antibodies with cross-reactive immunity to heterologous *Salmonella*, but exhibit less protective ability because not all antibodies elicited by OMPs bind effectively to the wild-type *Salmonella* due to the masking effect of the lipopolysaccharide layer on the surface of *Salmonella*. For example, OmpA, which is located as a monomer on the outer membrane of *Salmonella*, produces channels in the lipopolysaccharide layer that are not large enough to allow antibodies to cross the O-Ag into the bacterial surface for binding, and therefore antibodies against OmpA are not protective.^17^ However, certain conserved protein antigens on the outer membrane of *Salmonella* are immunoprotective because the footprint formed by these antigens is large enough for antibodies to readily cross the lipopolysaccharide layer. Such as OmpD in *S*. Enteritidis is nearly identical to the *S*. Typhimurium and differs only by a single amino acid, which is located on the outer membrane and exists as a trimer to generate a tunnel aperture size that allows a single Fab to cross the LPS channel and bind to the bacterial surface.^17^ Although only one Fab can access the LPS channel created by certain trimeric proteins in the surrounding LPS layer, it is also able to confer protection against infection of *Salmonella*. This explains our findings that cross-protection induced by the regulated delayed attenuated *Salmonella* is related to the level of effective antibody. Although we do not know which outer membrane proteins other than OmpD are capable of generating larger tunnels that allow antibodies to cross the O-antigen to bind to the outer membrane, we demonstrated that certain conserved antigens on the outer membrane do provide cross-protection to the challenge of *Salmonella*.

Theoretically, shorter LPS lengths of the attenuated *Salmonella* expose more OMPs and induce better immune responses against OMPs in mice, but in fact, after immunization with SW101 (*waaC*), SW105 (*waaF*), SW108 (*waaG*) mutants, whose C-OS consist of a partial or whole inner core in the absence of arabinose, mice did not show significant antibody responses against OMPs from *S*. Enteritidis or *S*. Choleraesuis (Figure 5). We speculated that this may be related to the slower transformation of SW101 (*waaC*), SW105 (*waaF*), and SW108 (*waaG*) to the rough phenotype in mice compared to SW114 (*waaI*), SW116 (*waaJ*), SW118 (*waaL*), and SW120 (*wbaP*), although we do not know the different regulated delayed attenuated *Salmonella* undergo the exact process of phenotypic transformation *in vivo*. The effect of the regulated delayed system on the expression levels of OMPs of attenuated *Salmonella* may also cause various immune responses induced by different attenuated *Salmonella* in mice (Supplementary Figure S3B). In addition to the surface OMPs, the inner and outer cores of different serotypes of *Salmonella* are conserved, and our experimental results showed that sera from SW118 (*waaL*) immunized mice not only recognize LPS of wild-type *S*. Typhimurium but can also specifically identify LPS from *S*. Enteritidis, and *S*. Choleraesuis (Supplementary Figure S10), implying that the *Salmonella* cores of LPS may also be one of the reasons why the mutants were able to induce cross-protective in mice. Although the SW108 (*waaC*), SW110 (*waaF*), and SW112 (*waaG*) strains failed to elicit considerably increased levels of IgG and IgA specific to OMPs from wild-type *S*. Enteritidis and *S*. Choleraesuis at 9 weeks after the initial immunization, C3 deposition on wild-type *S*. Enteritidis or *S*. Choleraesuis was also observed when we used sera from the SW108 (*waaC*), SW110 (*waaF*), or SW112 (*waaG*) immunized group (Figure 6a-c). This result may be due to the fact that the regulated delayed attenuated *Salmonella* induces antibody production specific to other conserved antigens of *Salmonella* in mice, such as the ECA, which is a carbohydrate-derived component that is conserved across all Enterobacteriaceae, and the exact role of the ECA is not well understood.

The vaccine administration induces the proliferation of long-lived plasma cells and memory B cells responsible for sustaining humoral immunity. In this study, it was observed that on days 72 and 120 after the initial immunization, the mice still exhibited significant levels of IgG antibodies. This finding suggests that the regulated delayed attenuated *Salmonella* effectively stimulated the production of a substantial population of long-lived plasma cells in the mice (Supplementary Figure S7), particularly in the case of SW118 (*waaL*). Furthermore, regulated delayed attenuated *Salmonella* can effectively stimulate the generation of B220^low^ IgG^+^ B_M_ in mice (Figure 8, Supplementary Figure S9) indicating that the regulated delayed attenuated *Salmonella* can successfully induce a persistent humoral immune response and maintain immune memory in mice.

In conclusion, we systematically investigated the immunogenicity and protective efficacy of delayed attenuated *Salmonella* mutants. Our results indicate that strains containing whole-core oligosaccharides of lipid A not only expose more conserved OMPs but also have the ability to elicit enhanced cross-protective immunity against both homologous and heterologous *Salmonella* infections.

## Supplementary Material

Supplemental Material

## Data Availability

The datasets used and/or analyzed during the current study to reproduce these findings are available from the corresponding author upon request.
