# Supplementary material for: Immunogenicity and cross-protective efficacy induced by delayed attenuated Salmonella with regulated length of lipopolysaccharide in mice
Source: Gut Microbes. 2024 Nov 11;16(1):2424983. doi: 10.1080/19490976.2024.2424983 (PMC11559367; doi:10.1080/19490976.2024.2424983)

**Supplementary Figure 1**. **The screening for mutations with tightly regulated LPS in a wild-type background.**

The TT *araC* P_BAD_ cassette with the candidate gene carrying two different SD sequences and/or start codons were integrated into the chromosome of UK1 in place of the *pagL* gene, respectively, to generate a series of mutants in which LPS synthesis is regulated by arabinose. The mutant strains were cultured in LB broth with or without 0.1% arabinose and then were carried out with silver stain. After the mutations with tightly regulated LPS were successfully screened and identified in the wild-type background, the correct mutation was introduced into the mutant strain SW067 (Δ*pagL7* Δ*pagP81*::P_lpp_ *lpxE* Δ*lpxR9* Δ*fur9*). The “+” indicates that arabinose is added to LB broth, while the “-” indicates that arabinose is not added to LB broth.


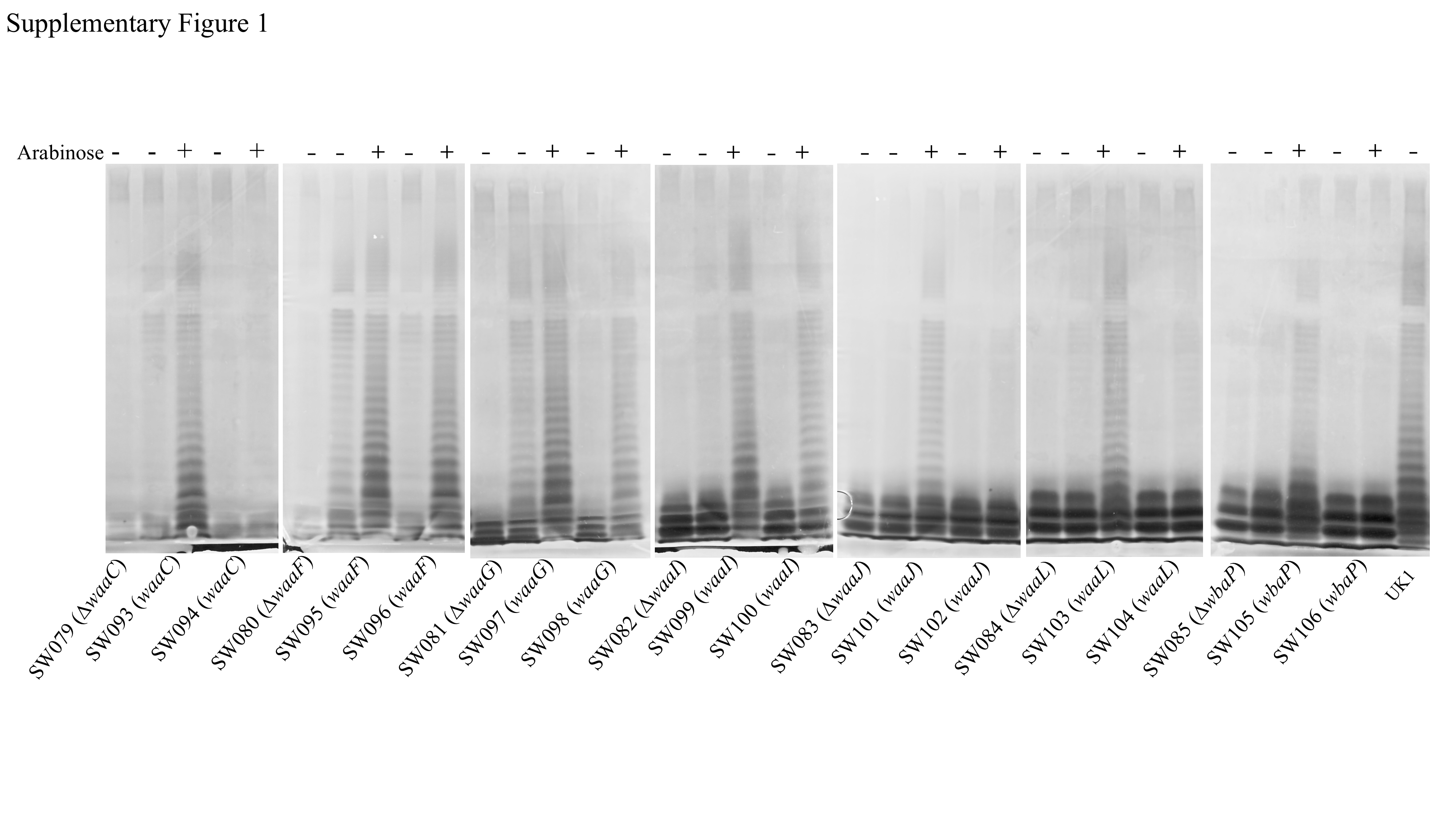


**Supplementary Figure 2**. **Phenotype determination of the mutant strains with the deleted glycosyltransferase genes.**

The genes *waaC*, *waaF*, *waaG*, *waaI*, *waaJ*, *waaL*, or *wbaP* were deleted from the genome of SW067 (χ9705 Δ*fur9*). The mutant strains were cultured in LB broth and then were performed with silver stain. The mutant strains exhibited an inability to produce a typical LPS banding pattern.


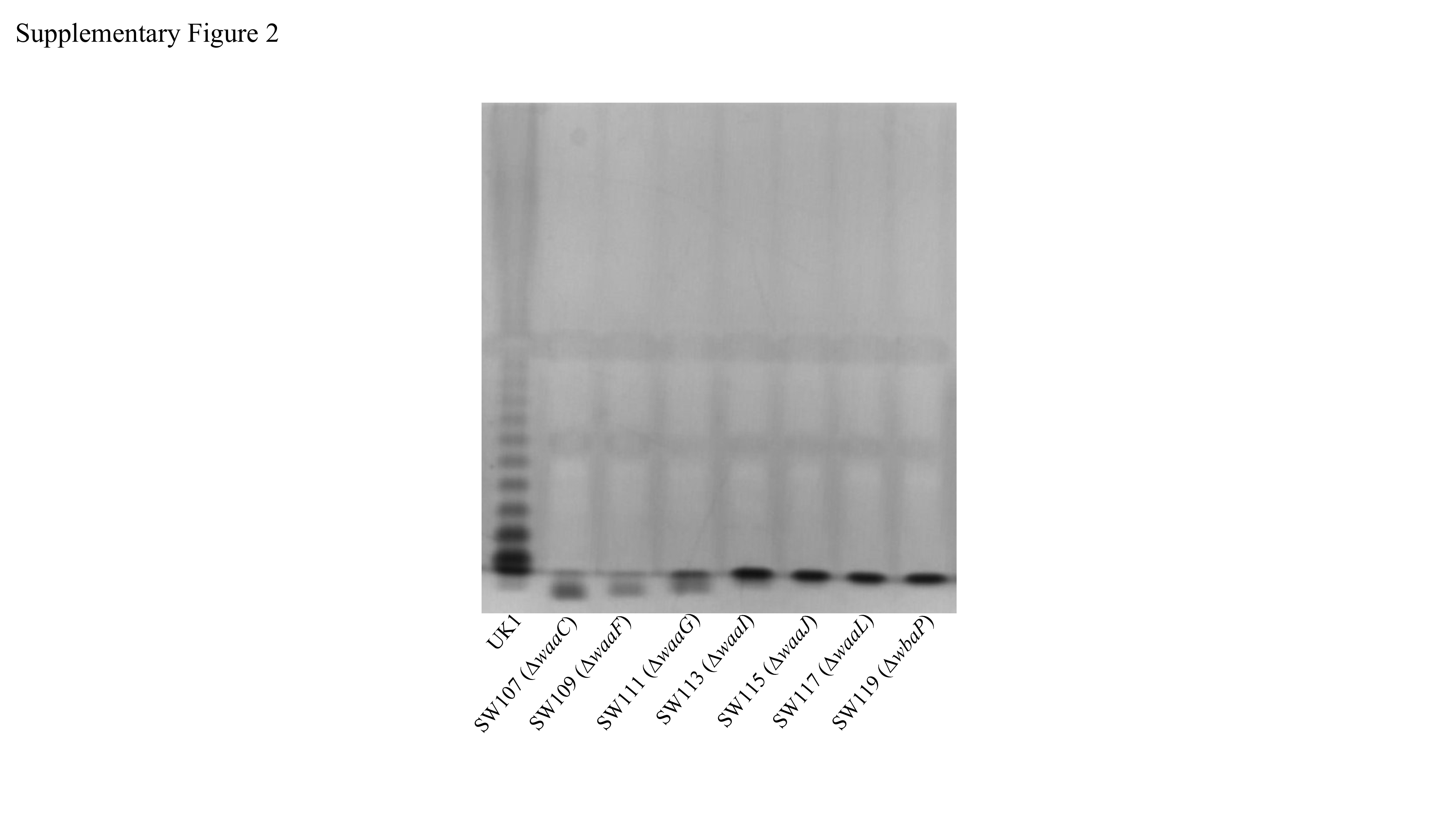


**Supplementary Figure 3. Phenotype determination of the regulated delayed attenuated *Salmonella*.**

(A) Mutants SW108 (*waaC*) was cultured in nutrient broth supplemented with different concentrations of mouse serum to construct silver-stained profiles, and UK1 was used as a control. (B) The OMPs from the mutants were isolated and analyzed on an SDS-PAGE gel staining by Coomassie Brilliant Blue. The “+” indicates that arabinose is added to the culture medium, while the “-” indicates that arabinose is not added to the culture medium.


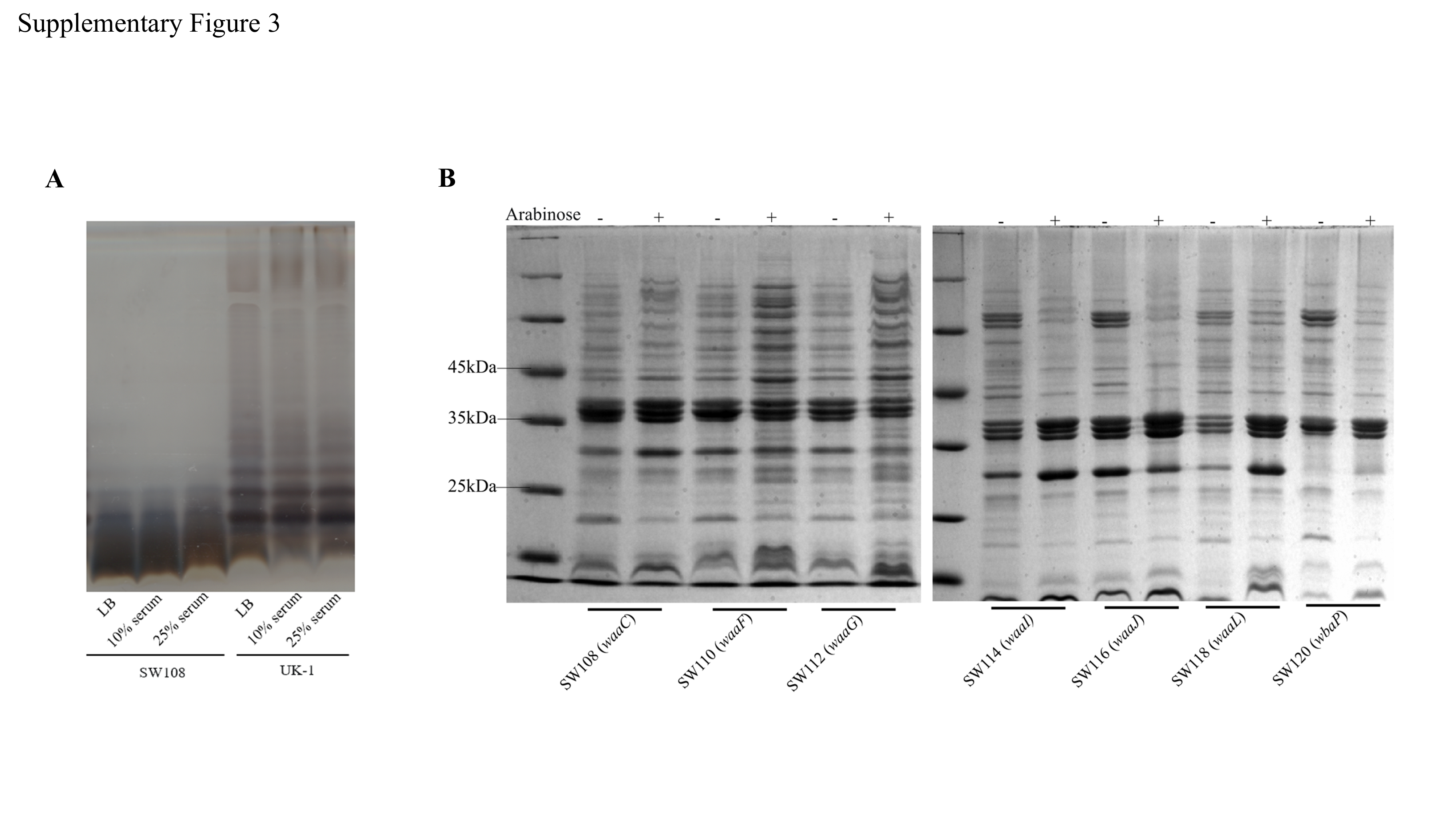


**Supplementary Figure 4. Bacterial burden in liver, spleen, Peryer’s patches of mice.**

The arabinose was added to the drinking water of the mice inoculated with SW108 (*waaC*) or SW118 (*waaL*), and we evaluated the bacterial loads in the spleen, liver, and Peyer′s patches of mice on days 3, 6, 14, and 21 after the initial immunization. The “+” indicates that arabinose is added to the drinking water, while the “-” indicates that arabinose is not added to the drinking water. The standard differences between the mice in each group were shown by the error bars. Data are presented as the means ± SEM (n = 3). The superscript letters a indicate P < 0.05 for comparisons with the SW118 (*waaL*) group, in which arabinose is not added to the drinking water. The dotted black line indicates the minimal detection limit of the test (10 CFU/g).


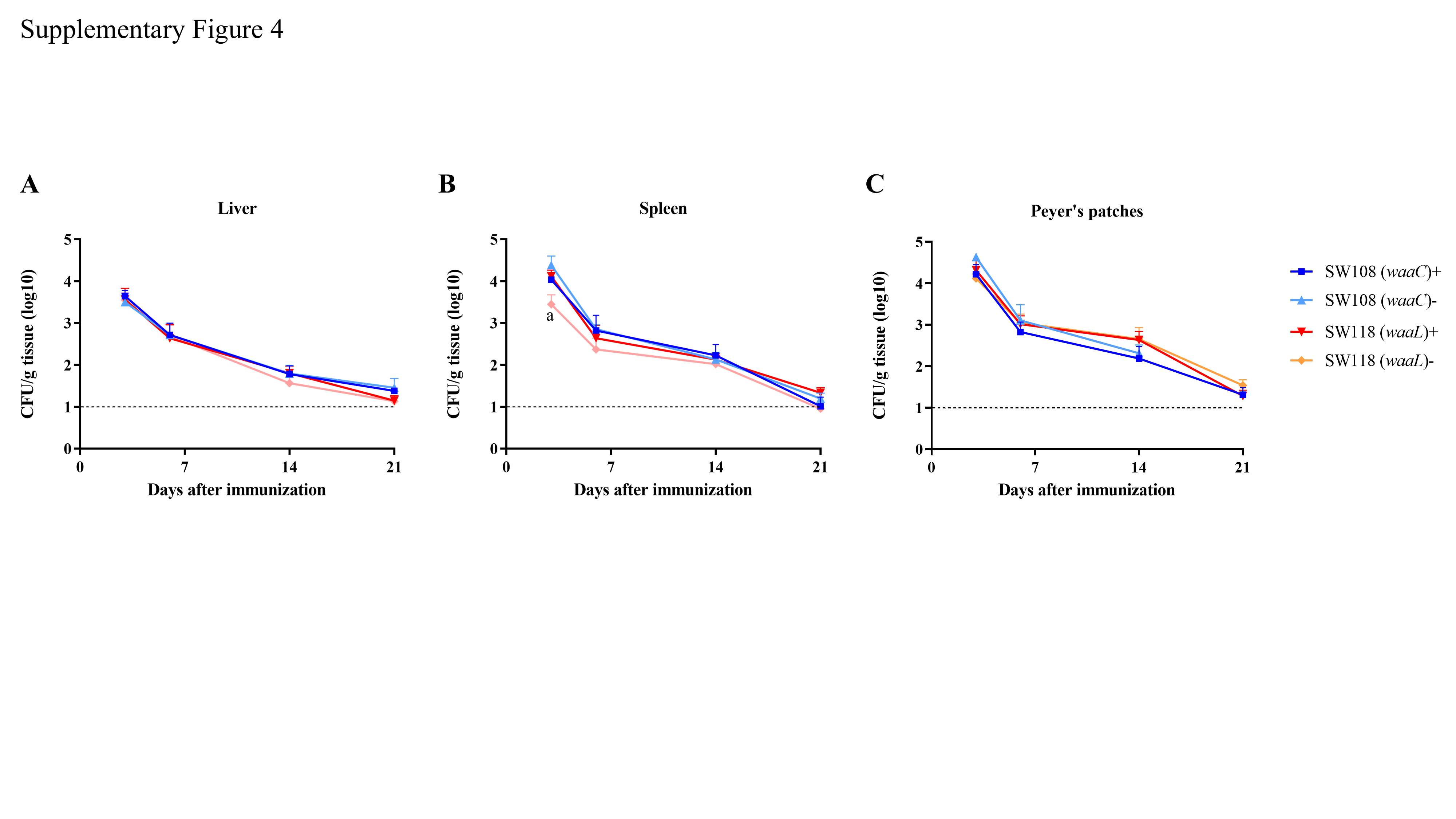


**Supplementary Figure 5. The gating strategy of CD3^+^ CD4^+^ and CD3^+^ CD8^+^ T cells.**

The percentage of CD3^+^ CD4^+^ and CD3^+^ CD8^+^ T cells from immunized mice was evaluated by flow cytometry on day 38 after the initial immunization. The representative dot plots derived from the flow cytometry analysis showed the gating strategy, including lymphocytes > singlets > live cells > CD3^+^ T cells > CD3^+^ CD4^+^/CD3^+^ CD8^+^ T cells.
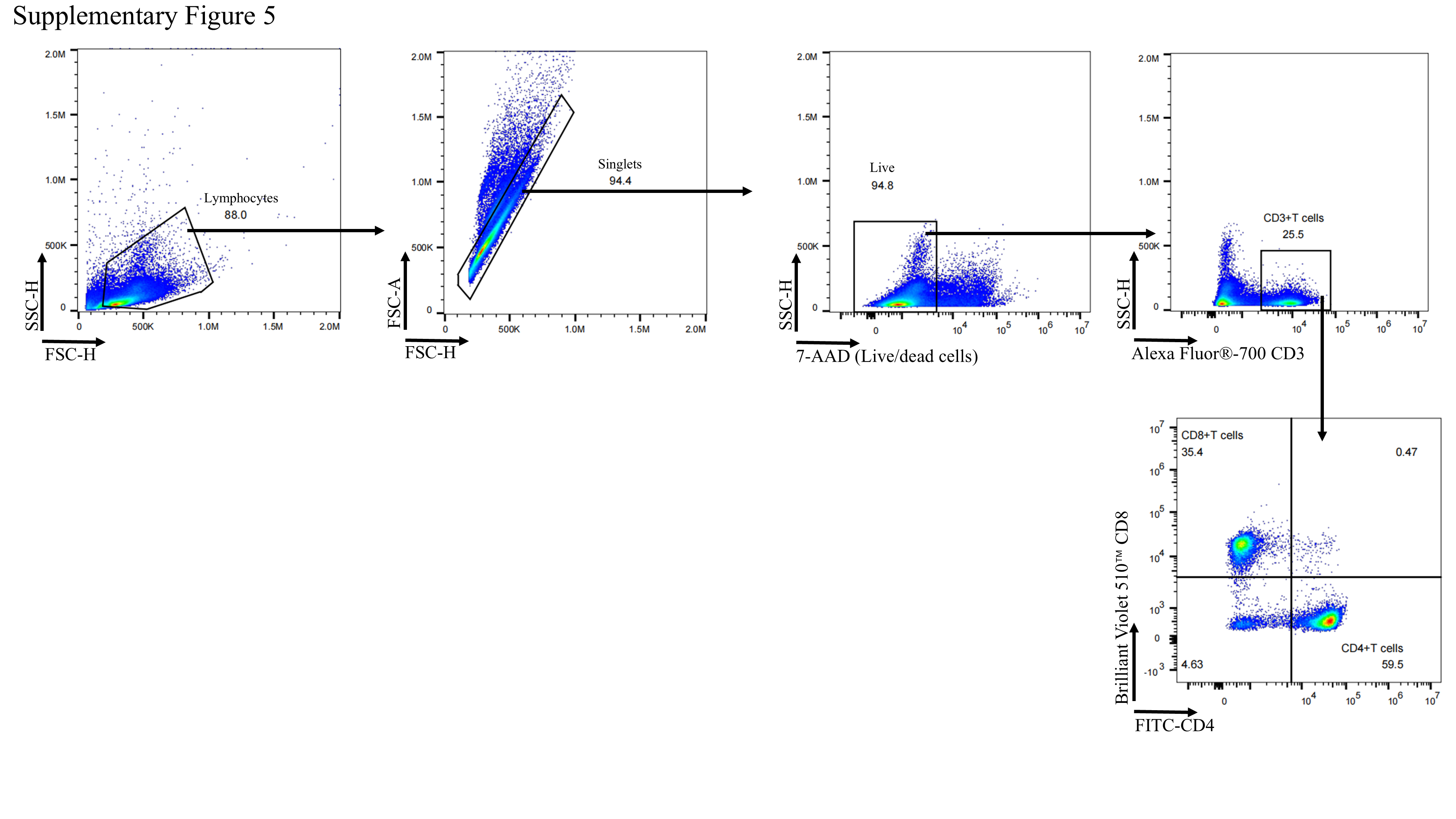


**Supplementary Figure 6. Determination of the proportion of CD3^+^ CD4^+^ and CD3^+^ CD8^+^ T cells.**

The percentage of CD3^+^ CD4^+^ and CD3^+^ CD8^+^ T cells from immunized mice was evaluated by flow cytometry on day 38 after the initial immunization. Dot plots derived from the flow cytometry analysis from experiments with all samples per group.


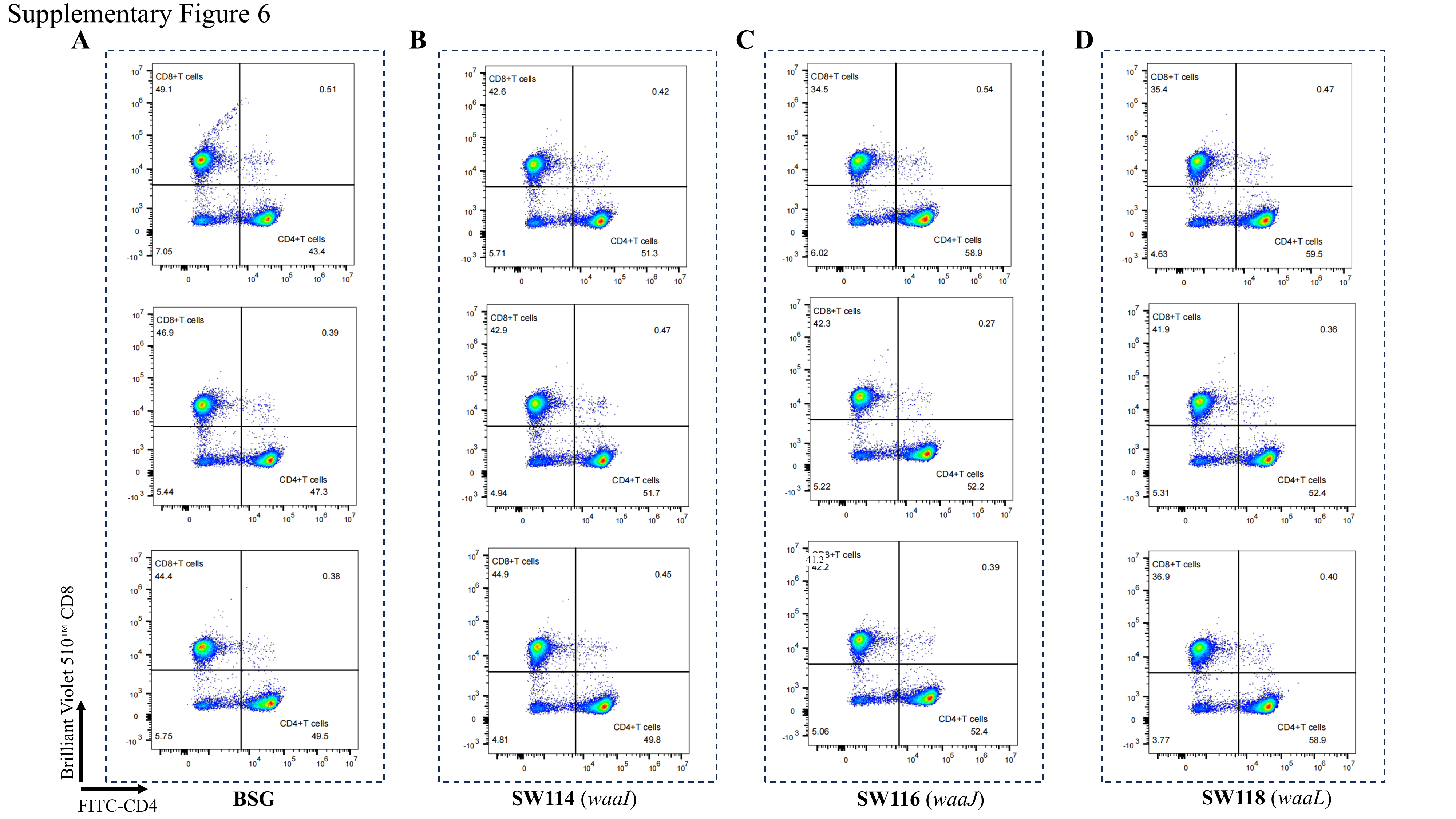


**Supplementary Figure 7. The assessment of long-term** **immune responses induced by regulated delayed attenuated *Salmonella*.**

The sera were gathered from mice in SW114 (*waaI*), SW116 (*waaJ*), and SW118 (*waaL*) groups on days 72 and 120 after the primary immunization. Quantitative ELISA was applied to analyze the level of IgG specific to OMPs from *S*. Typhimurium (A, D), *S*. Enteritidis (B, E), and *S*. Choleraesuis (C, F) on days 72 and 120. The results displayed the precise levels of antibodies, as measured by a standard curve. The standard differences between the mice in each group were shown by the error bars. Data are presented as the means ± SEM (n = 8), superscript letters a, b, and c indicate P < 0.05 for comparisons with the BSG, SW114 (*waaI*), and SW116 (*waaJ*) groups, respectively.


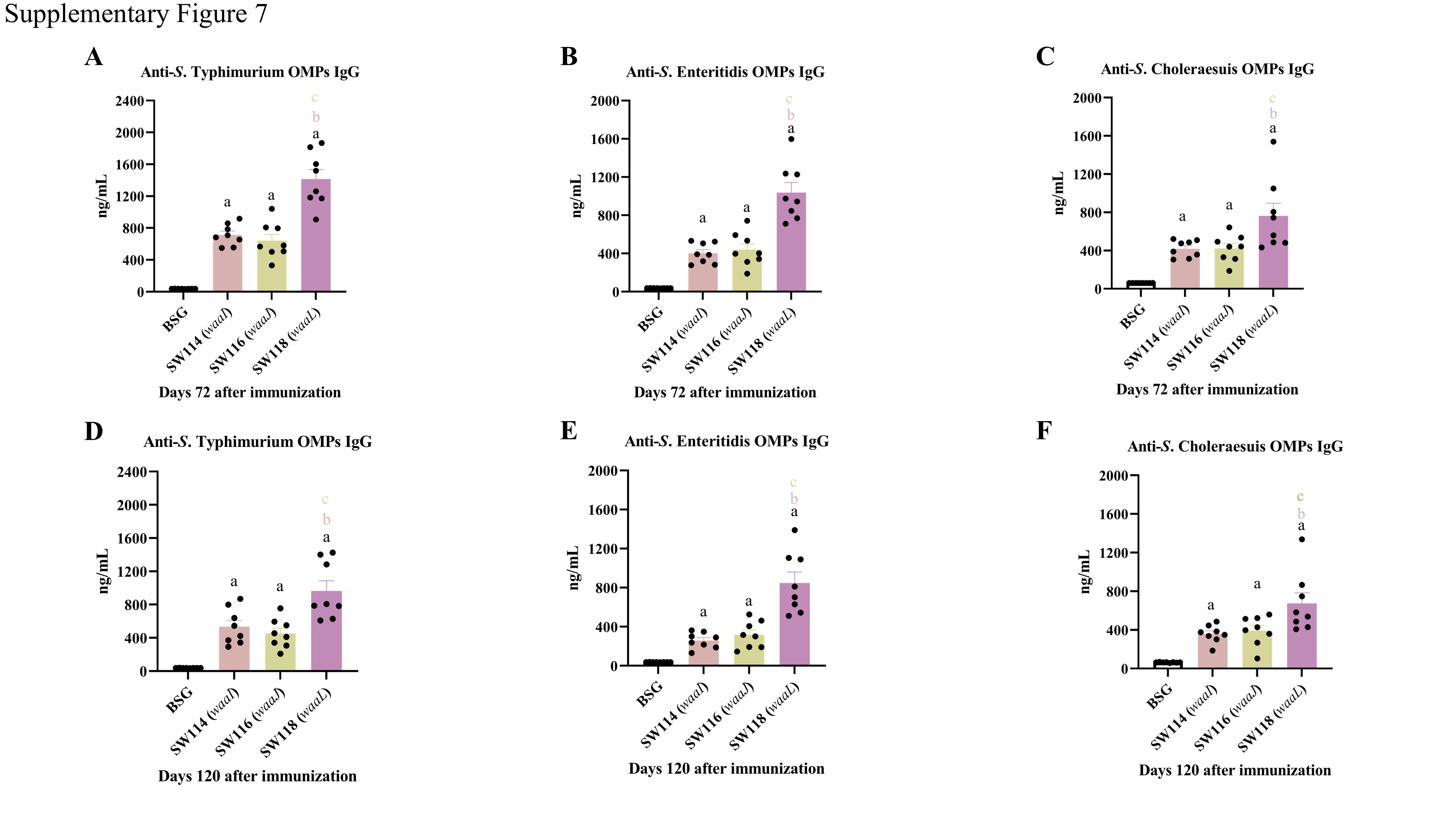


**Supplementary Figure 8. The gating strategy of B220^low/int^ CD80^+^ IgD^-^ IgG^+^ B_M_ cells.**

The percentage of B220^low/int^ CD80^+^ IgD^-^ IgG^+^ B_M_ cells from immunized mice was evaluated by flow cytometry on day 72 after the initial immunization. B_M_ cells were gated as B220^low^ CD80^+^ IgD^-^ IgG^+^ cells. The representative dot plots derived from the flow cytometry analysis showed the gating strategy, including lymphocytes > singlets > live cells > B220^low^/B220^high^ B cells > CD80^+^ B cells > IgD^-^ B cells > IgG^+^ cells.


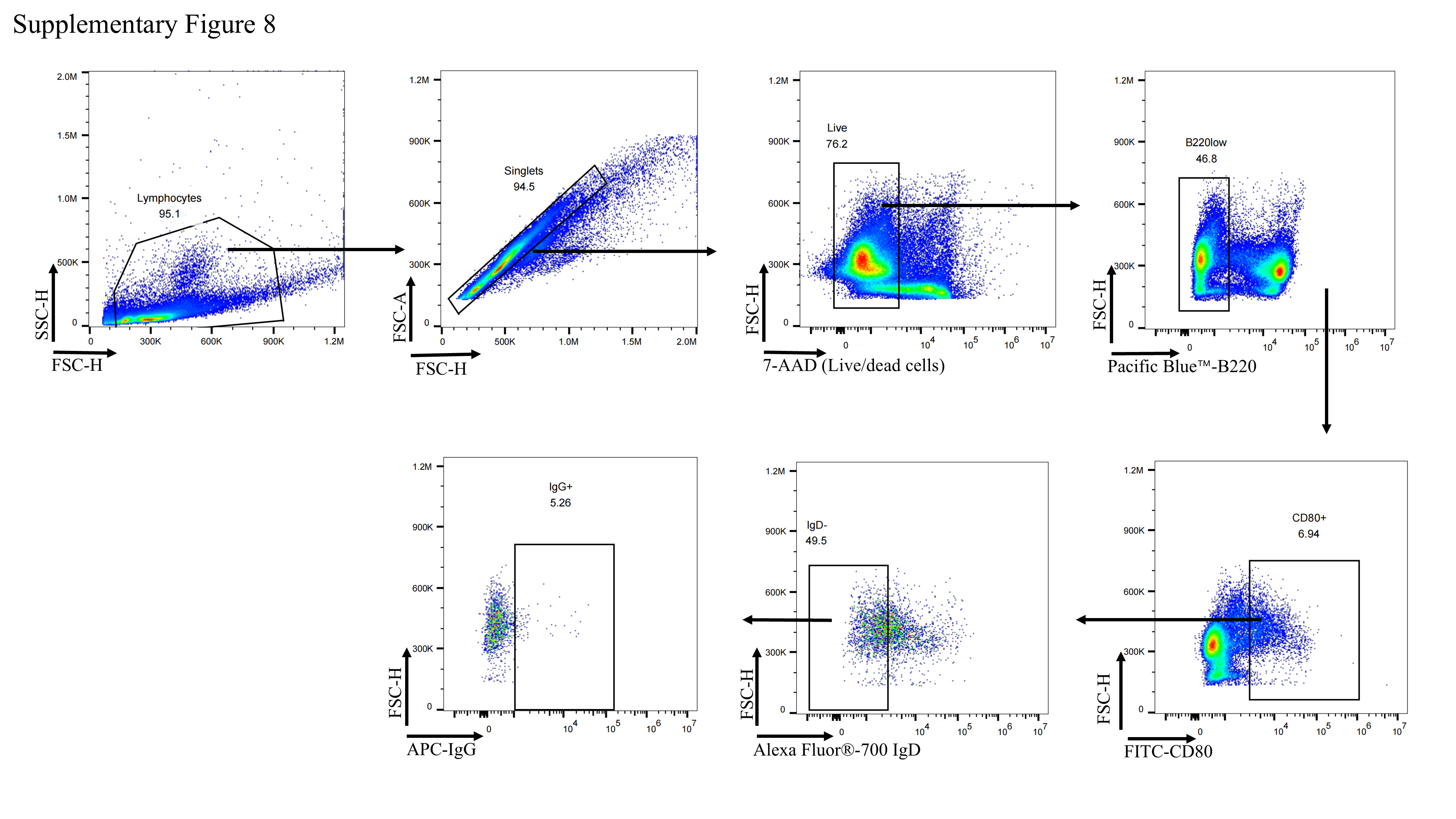


**Supplementary Figure 9. Determination of the percentage of IgG^+^ B_M_ cells.**

The percentage of B220^low/int^ CD80^+^ IgD^-^ IgG^+^ B_M_ cells from immunized mice was evaluated by flow cytometry on day 72 after the initial immunization. B_M_ cells were gated as B220^low^ CD80^+^ IgD^-^ IgG^+^ cells. Dot plots derived from the flow cytometry analysis from experiments with all samples per group.


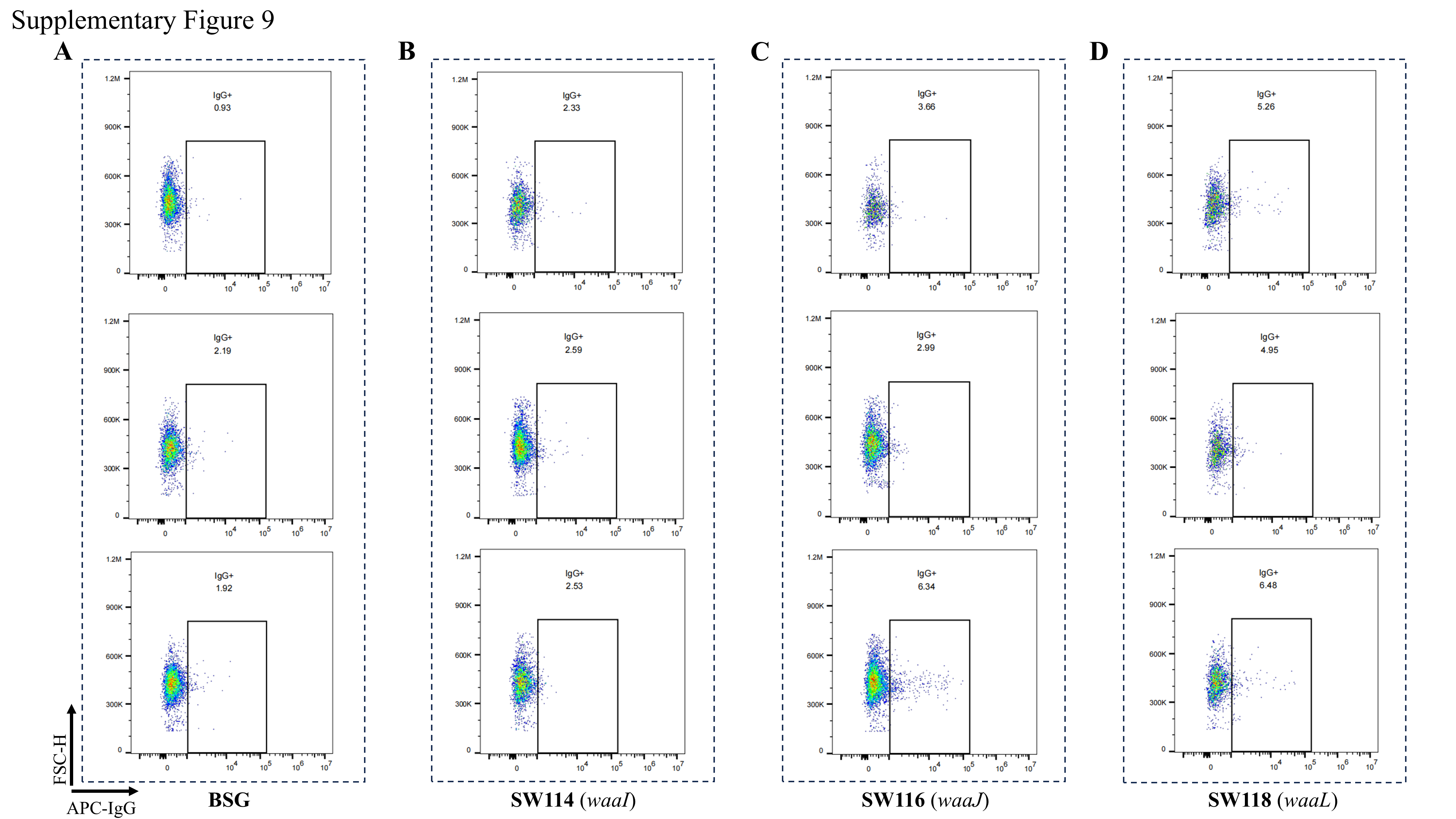


**Supplementary Figure 10. The assessment of immune responses against *S*. Typhimurium, *S*. Enteritidis, or *S*. Choleraesuis LPS induced by regulated delayed attenuated *Salmonella*.**

The sera were gathered from immunized mice on days 56 after the primary immunization. The levels of specific IgG antibodies to *S*. Typhimurium (A), *S*. Enteritidis (B), or *S*. Choleraesuis (C) LPS were detected by quantitative ELISA. The results displayed the precise levels of antibodies, as measured by a standard curve. The standard differences between the mice in each group were shown by the error bars. Data are presented as the means ± SEM (n = 8), superscript letters a indicate P < 0.05 for comparisons with the BSG groups.


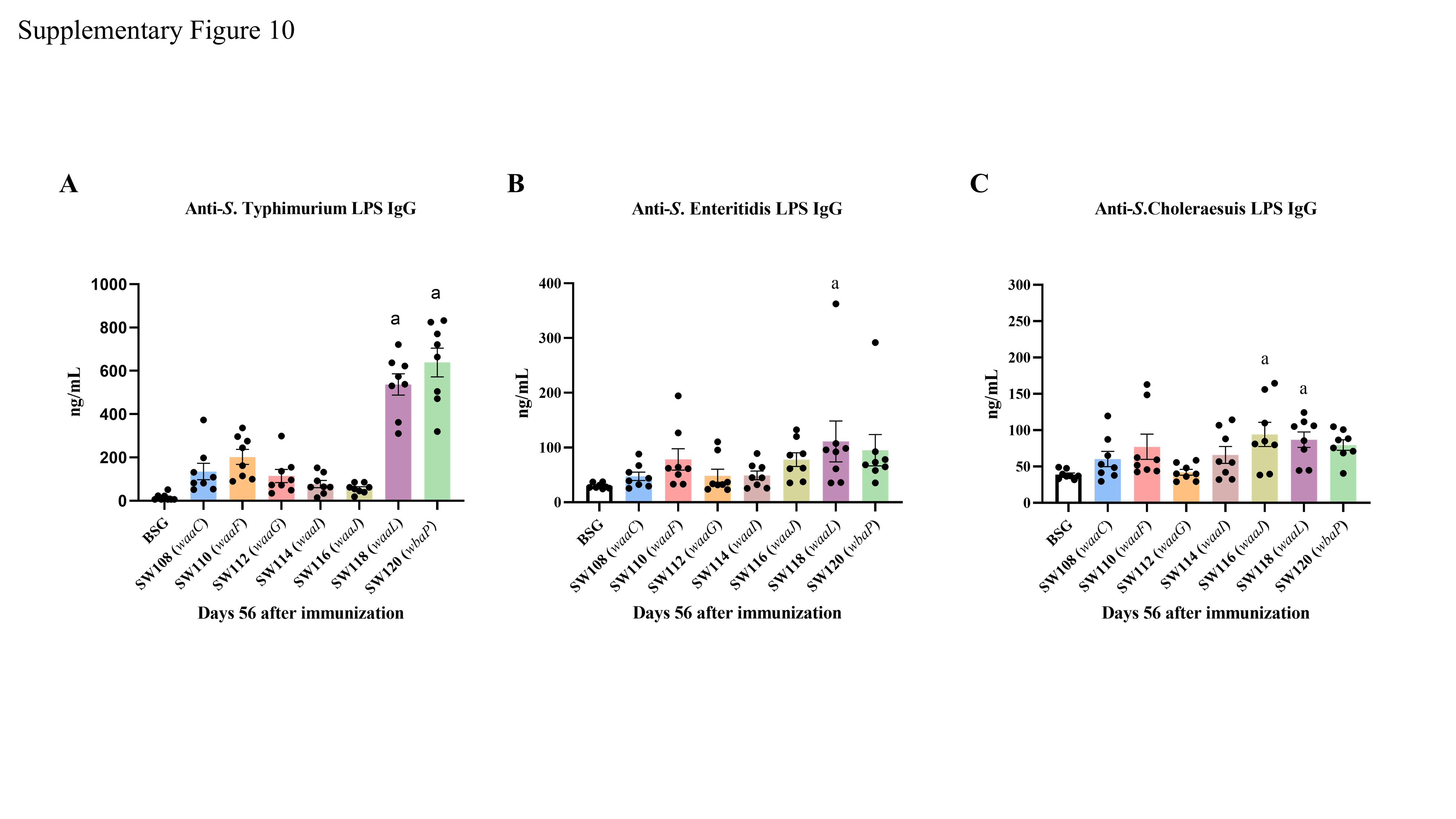

Supplement: Supplemental Material [file KGMI_A_2424983_SM2509.docx]
